# Effect of households’ members disability and serious illness on public health insurance subscription among urban refugees during the COVID-19 pandemic in Kenya

**DOI:** 10.1186/s12889-024-20794-1

**Published:** 2024-11-26

**Authors:** Abayomi Samuel Oyekale, Thonaeng Charity Molelekoa

**Affiliations:** Department of Agricultural Economics and Extension, North-West University Mafikeng Campus, Mmabatho, 2735 South Africa

**Keywords:** Disability, Serious illness, Health insurance, Adverse selection, COVID-19, Refugees, Urban Kenya

## Abstract

**Background:**

The adverse selection theory speculates a high level of demand for health insurance by people with vulnerable health conditions. However, the COVID-19 pandemic changed the prevailing narratives and pattern of healthcare utilization in many African countries. This study estimated the effects of household member’s disability and presence of serious illness on the probability of National Hospital Insurance Fund (NHIF) subscription with the average treatment effect (ATE) and average treatment effect on the treated (ATET).

**Methods:**

The data were collected telephonically in 2020 using the sampling frame of the United Nations High Commission on Refugees (UNHCR). The respondents were refugees with active phone numbers who were registered by the UNHCR in Nairobi, Mombasa and Nakuru cities. A total of 2,438 completed the surveys. The data were analysed with Treatment Effects Probit regression model using the regression adjustment estimator.

**Results:**

The results showed that 24.89% of the respondents had health insurance. Also, 3.28%, 1.39% and 2.46%, respectively suffered from physical, cognitive and sensory disability, while 8.28% had some form of serious illness. The Probit regression results showed that probability of being health insured significantly increased (*p* < 0.05) with membership of community-based organizations (CBO), asset index, possession of bank savings account, residence in Nairobi and household size, while residence in Nakuru reduced it. The ATE for physical and cognitive disabilities were significant (*p* < 0.05) with 0.1100 and 0.1816, respectively, while that for serious illness was 0.1046 (*p* < 0.01). The ATET for physical disability and serious illness were also significant (*p* < 0.05) with 0.1251 and 0.0996, respectively.

**Conclusion:**

It was concluded that efforts to facilitate NHIF subscriptions among the refugees should be channelled among people with disability and serious illness. In addition, there is the need to promote refugees’ welfare through employment that can induce formal savings and promote less reliance on informal borrowing. The operational mechanisms and differences in healthcare service distribution between the three cities should be considered along some salient interventions for health insurance subscription that are channelled through some CBOs.

**Supplementary Information:**

The online version contains supplementary material available at 10.1186/s12889-024-20794-1.

## Introduction

Inadequate access to healthcare services is one of the major deprivations suffered by refugees across the world. The welfare of refugees can no longer be subjected to political games, given that recent statistics have shown that as of mid-2024, 122.6 million people across the globe were forcibly displaced [1]. It is further disheartening to note that given the current impacts of anthropogenic climate change, more than 200 million people would be on the move by 2050 [2]. The global empathy for a restructured health system that caters for the medical needs of everyone has been amplified by the COVID-19 pandemic. Specifically, the pandemic produced an offshoot of preventive interventions that completely disregarded the plights of displaced people, as human movements were restricted and international borders were indefinitely closed [3].

Although the tenet of Universal Health Coverage (UHC) remains the slogan of our contemporary healthcare service administration and delivery [4, 5], persistent inequality in the coverage and quality of healthcare services still exists with refugees being the most deprived [6, 7]. In many instances, the condition of healthcare service facilities in refugees’ camps depicts a rude narrative of neglect and complete forgetfulness of the fact that access to quality healthcare services remains a fundamental right of everyone [8]. Therefore, although the primary agenda of the Sustainable Development Goals (SDGs) is to ensure that no one is left behind in terms of affordability of the means to a dignified life, growing poverty and persistent inequality often refute the seriousness and sincerity of development policymakers. It should be noted that whichever way the die is cast, in the context of development planning, access to healthcare services is one of the foremost human rights that has been politically compromised.

Furthermore, it should be noted that majority of refugees in the world reside in developing countries [9, 10], where existing healthcare facilities may be inadequate for the citizens. In addition, at the end of October 2023, there were 676,332 registered refugees and asylum seekers in Kenya [11]. Majority of them originated from Somalia, Uganda, and South Sudan, having fled from insurgencies, food insecurity and environmental degradation [12]. This number increased to 777,654 as of 30th June 2024 [13], and placed Kenya as the fifth largest host of refugees in Africa [14]. In addition, about 16% of these people reside in urban areas, which offer some benefits of employment opportunities and access to basic social services when compared to their counterparts in camps [15]. Kenya’s humanitarian benevolence to refugees had evolved from restrictive to integration policies. Specifically, the Kenyan Government constitutionally signed the Refugee Act 2021 into law, with the primary aim of promoting their economic inclusiveness [16].

The rising number of refugees in Kenya also places some extra demand on the social services, especially the healthcare infrastructure. Therefore, a proper understanding of refugees’ health needs is essential to promote effective planning towards attainment of some SDGs. It should be noted that Kenya has 9,696 registered health facilities, of which 4,616 are publicly owned, 3,696 are private-for-profit, and 1,384 are Faith Based Organizations (FBOs)/Non-Governmental Organizations (NGOs)/Community Based Organizations (CBOs) [17]. Since 2005, the Primary Health Care (PHC) service delivery in Kenya has been driven by Kenya Essential Package for Health (KEPH) concept, which identifies disease hot spots, age cohort cost effective interventions, and the nature of service package [18]. Over the years, the need for health insurance as a means for timely access to healthcare services has been realized. This led to the 1966 Act of Parliament that set up the National Health Insurance Fund (NHIF) as an integrated Department in the Ministry of Health. In 1998, the NHIF evolved into a State Corporation under the NHIF Act No 9 [19]. With membership opened to all Kenyans 18 years and above, the major mandate of the NHIF is to ensure adequate medical insurance for all subscribers and their registered dependants. The Fund comprised of ninety-five branches, which are fully autonomous, satellite offices and a presence in forty-seven “Huduma Centers” [19].

However, there are currently 75% of Kenyans who are not health insured, thereby depending on out-of-pocket payments [20]. Recent influx of refugees to Kenya places unprecedented pressures on the health system. This implies that a proper understanding of health insurance uptake among refugees is essential to evaluate their degree of avoiding some catastrophic health expenditures [20]. Although many refugees are healthy [21], a good number of them may also suffer from some health challenges [7, 22]. Specifically, different factors influence their health status upon arrival at their destination countries. These include the magnitude of insecurity and stress of the journey, initial health condition at the country of origin, and immediate access to financial and social support [23–25]. In their unique heterogeneity, the refugee population represents a culturally diverse people with peculiar healthcare needs and challenges [26].

Conceptually, the healthy migrant effect denotes a situation where refugees possess better health condition than their host communities upon arrival [27]. However, the passage of time will nullify the initial differences due to several barriers in relation to healthcare access, cultural restrictions, language differences, and access to labour markets [28]. Refugees’ level of health literacy is a fundamental factor explaining their health status due to the mediating influences of treatment compliance, adequate nutrition, inactivity, and psychological wellness [29]. The cumulative impacts of health risk exposures and their management operations will comprehensively determine the refugees’ stock of health, which in some worse scenarios may manifest in disability and serious illness [29]. Furthermore, the health needs of refugees’ children can be significantly compromised by inadequate nutrition, thereby increasing their risks of adverse health outcomes [26, 30–33]. Moreover, inadequate attention to refugees’ sexual and reproductive health can compromise their well-being through sex-related infections, unwanted pregnancy [34], and a very high risk of deteriorated health among pregnant women [26]. Refugees’ health responses through adequate nutrition, timely treatment and effective identification of underlying vulnerability may reduce the progression of some mental stressors [35, 36] into disability and serious illness.

In some other instances, the significance of social factors influencing health was emphasized by the World Health Assembly in 2009, 2012 and 2021 [37]. These factors include gender, education level, social assistance, households’ income, nature of employment, nutrition status, housing characteristics, healthcare accessibility and affordability, and conflict [3]. Some studies have also highlighted the role of food insecurity in the promotion of depression and nutrition-related disability [38–40]. The cumulative impacts of socioeconomic deprivations often result into disability and illness [41], thereby increasing the need for medical services. The adverse selection theoretics have therefore emphasized the tendency of people with fragile health conditions in opting for health insurance, while moral hazard emphasizes the tendency of the insured individuals to act carelessly since they are insulated from the full consequences of their actions. The adverse selection theory was introduced by Akerlof [34] and it depicts a kind information asymmetric between health insurers and the insured. Therefore, avoidance of out-of-pocket catastrophic expenses can compel people with disability or serious illness to subscribe to some form of health insurance [42].

It should be acknowledged that although several studies had been done on the determinants of health insurance subscription, only very few had focused on refugees’ population. This can be traced to data paucity and some legislative restrictions preventing refugees from being fully integrated into accessing some frontline social services in their host countries. A study by Quartey [43] among Ghana residents who were at the risk of statelessness found education, being married and young to promote health insurance uptake, while joblessness reduced it. Oraya [44] also found that health insurance uptake among Kenyan refugees was promoted by education, age, access to information and marital status. A study by Dias et al. [45] found utilization of healthcare services among immigrants in Portugal to be influenced by the country of origin and the years already stayed.

Kimani et al. [46] found that health insurance subscription among Kenyan women was positively influenced by formal employment, being married, access to media information, wealth index, and female headship, while residence in the central and northeastern regions reduced it. Kazongu and Barasa [47] found that the probability of health insurance subscription increased among older people, the formally employed, the married, media exposed, males, people living with serious disease, the wealthy, and small household sizes. Kimani et al. [48] also found that among urban slum residents in Kenya, the likelihood of health insurance subscription increased significantly with membership of organizations, formal employment, and marital status.

This study contributes to existing literature in different ways. First, from the theoretical lens of adverse selection, the study presents the correlates of health insurance subscription and analysed the effects of disability and serious health problems on health insurance subscription among refugees using the regression adjustment estimator. In addition, while studies on refugees in camps are predominant, few studies exist on urban refugees. Specifically, a comprehensive understanding of the health insurance subscription behaviour of urban refugees will facilitate our understanding of the extent of current level of healthcare deprivations and the need for interventions to promote equity. This study therefore bridges some existing gaps in the health insurance literature by estimating the health insurance effects of disability and serious illness among urban refugees in Kenya with the ATE and ATET indicators.

## Materials and methods

### The data and sampling procedures

The data were collected by the United Nations High Commissions on Refugees (UNHCR) in 2020 as part of the initiatives to understand the socioeconomic impacts of the COVID-19 pandemic on urban refugees. This study used a case control research design due to some ethical issues underpinning random allocation of people into any of the treated groups. This design collects information from a set of individuals at a point in time without influencing any of their characteristics [49]. The UNHCR ProGres register of urban refugee households was used as the sampling frame. This register groups refugees into families, which may not necessarily be households eating from the same pot. Therefore, the definition of family is contrary to the conventional definition of households as being used in many cross-sectional surveys. Within the families, the households were classified. Therefore, the survey progressed with sampling of families and subsequent identification of the households to which they belonged.

The questionnaire was designed in English, and it was divided into twelve sections. The informed consent section ensured a voluntary participation by every respondent, who must be the head or an adult member of the selected household. Moreover, the other sections of the questionnaire were derived from validated questionnaires which had been previously used for several socioeconomic surveys among refugees [50]. The sampling frame comprised of those refugees with active phone numbers. Therefore, telephone interviews were conducted due to several COVID-19 social distancing and movement restrictions. The enumerators were trained to ensure ethical compliance and consistency of the generated data. Moreover, in cases where the respondent could not properly communicate in English language, multilingual enumerators were engaged to translate the questionnaire into several foreign languages.

The survey was conducted in the cities of Mombasa, Nairobi and Nakuru. These cities are compatible in terms of their phone penetration rates with Mombasa and Nairobi having 93% and Nakuru had 95%. Although 2500 respondents were needed for a less than 5% margin of error, 95% confidence level, and at least 50% population representation, 2438 households eventually completed the survey. The data collection lasted between 1st of November and 31 December, 2020. The stratified random sampling was used, and each of the selected cities was a stratum. Samples were allocated to each stratum in proportion to the number of registered refugee households. Precisely, a total of 729, 409 and 1300 households completed the surveys through the Computer Assisted Telephone Interview (CATI) in the cities of Mombasa, Nakuru and Nairobi, respectively.

### Limitation to the study

Although sampling weights were generated to enhance data representativeness, some limitations on the dataset should be highlighted. First, in statistical analyses, the results from the data for urban refugees cannot be compared with those for host communities due to slight differences in the method of questionnaire administration. In addition, this survey did not collect data on consumption to reduce the length of time for telephone interviews [15]. Therefore, consumption data, which could have been used as proxy for incomes are completely absent. Finally, the survey was only representative of those with phone numbers, and not necessarily for the entire urban refugee population.

## Methods of data analyses

### Principal component analysis (PCA)

PCA is a statistical tool for data reduction [51]. Therefore, we used it to aggregate some data variables into a composite indicator to reduce the number of independent variables and prevent unnecessary introduction of multicollinearity into the proposed outcome model. Specifically, three variables were generated with PCA, vis-à-vis, housing deprivation index, asset index and food insecurity coping index. The housing deprivation index was computed from 10 variables, which were dwelling type (unfinished or homeless = 1, 0 otherwise), floor materials (earth/sand or dung or plank/palm/bamboo = 1, 0 otherwise), roof materials (grass or dung or tin cans = 1, 0 otherwise), wall materials (unfinished = 1, 0 otherwise), run out of water (yes = 1, 0 otherwise), drinking water (unimproved = 1, 0 otherwise), toilet (unimproved = 1, 0 otherwise), sharing toilet (yes = 1, 0 otherwise), lighting energy (unimproved = 1, 0 otherwise), and cooking energy (unimproved = 1, 0 otherwise). The asset index was computed by coding ownership of each of the following 20 assets as 1 and 0 otherwise: radio, television, satellite dish, smartphone, refrigerator, table, mattress, mosquito nets, fan, bicycle, motorcycle, car, generator, solar panel, kerosene stove, charcoal stove, wheelbarrow, iron fencing, and chicken/other livestock. Food insecurity coping index was computed from the following nine variables coded as 1 for yes and 0 otherwise: sold households assets/goods, reduced spending on health or education, sold productive assets or means of transport, spent savings, borrowed money/food from a formal lender or bank, sold a house or land, withdrew children from school, sold the last female animal, begged and sold more animals than usual.

### Probit regression

The effects of disability and serious health problems on the probability of being health insured was analysed using the treatment effects framework provided by STATA 18 [52]. This framework does not require experimental data because it was designed for observational data where control and treatment groups may not have been randomly assigned. The analysis was implemented with STATA 18 software, using the regression adjustment estimator. The implementation requires specification of the correlates of the outcome variable, which is uptake of health insurance. In addition, due to the binary nature of the dependent variable, the Probit regression model was used with those who subscribed to the NHIF coded 1 and 0 otherwise. We have specified the model as follows:

1 $$Y_i=\alpha+\sum_{k=1}^{17} \beta_k X_i+\epsilon_i$$

$\:{Y}_{i}$ is the insurance subscription variable, $\:{\beta\:}_{k}$ and $\:{\alpha\:}_{i}$ are the estimated parameters, $\:{X}_{i}\:$are the explanatory variables and $\:{ϵ}_{i}$ is the error term. The coding formats of the explanatory variables are as shown in Table 1.

**Table 1:** Description of variables and their coding formats

Variables	Coding format
NHIF subscription (dependent)	Yes = 1, 0 otherwise
Community Based Organization membership	Yes = 1, 0 otherwise
Bank loans access	Yes = 1, 0 otherwise
Community savings loans access	Yes = 1, 0 otherwise
Family, relative, friend loans access	Yes = 1, 0 otherwise
Village savings participation	Yes = 1, 0 otherwise
Bank savings participation	Yes = 1, 0 otherwise
MPESA savings participation	Yes = 1, 0 otherwise
Pillow savings participation	Yes = 1, 0 otherwise
Separated child in living in household	Yes = 1, 0 otherwise
Person 60 and above in the household	Yes = 1, 0 otherwise
Pregnant or nursing mother in the household	Yes = 1, 0 otherwise
Standard of living index deprivation	Index Computed with PCA
Asset index	Index Computed with PCA
Food coping index	Index Computed with PCA
City of Residence (Mombasa City is the Reference)	
Nairobi City Resident	Yes = 1, 0 otherwise
Nakuru City Resident	Yes = 1, 0 otherwise
Household size	Number

### Computation of the ATE and ATET

We evaluated the effects of the treatment variables, which are presence of household member(s) with disability and serious illness. In the questionnaire, three variables were used to capture disability, and they are physical disability, cognitive disability and sensory disability. However, no specific identification of the nature of serious illness was made in the questionnaire. In the literature, serious illness encompasses those health conditions that affect the quality of life of the affected person over a long period of time, that may end in death irrespective of how long it takes, or that require periodic hospitalization [53]. These variables were each coded as 1 for those who responded yes and 0 otherwise. We estimated two parameters to deduce the differences in average probability of subscription to NHIF. The first is the Average Treatment Effect (ATE), which shows the difference between the expected outcomes for the treated and control groups [$\:E\left({y}_{1}-{y}_{0}\right)]$. The second parameter is the Average Treatment Effect on the Treated (ATET), which shows the difference between the average outcomes for the treated when they were treated, and their counterfactuals ([$\:E\left({y}_{1}-{y}_{0}\right)/t=1]$.

## Results

### Refugees’ socioeconomic characteristics

The results in Table 2 show the descriptive statistics of selected urban refugees’ demographic variables in Kenya. The Table shows that 54.38% of the respondents were males, while average age was 37.31 years. In addition, the average household size was 3.55. Based on countries of origin, Somalia and Democratic Republic of Congo dominated with 34.11% and 28.77%, respectively.

The Table shows that 24.9% of the respondents subscribed to the NHIF. The results also showed that 7.4% were members of CBOs. Also, 40% of the respondents were utilizing loans from family members/relatives/friends, followed by 1.1% who used informal loans and 0.9% who used bank loans. Based on the different types of savings, it was revealed that majority (55.6%) of the respondents had MPESA savings, followed by 17.7% that had pillow savings, 2.7% that had their savings in the bank and 0.9% that had village savings.

Table 2 further revealed that 3.4% of the households had children who were separated from their families. The disability/serious illness aspects of the results showed that 8.28% of the respondents indicated presence of member(s) suffering from serious illness. Moreover, 3.3% of the households had members who suffered from physical disability, 2.5% had members who suffered from sensory disability, and 1.4% had members with cognitive disability. It was further revealed that 7.3% of the respondents had member(s) who was (were) at least 60 years of age. The results also showed that 4.7% of the households had member(s) who was(were) either pregnant or nursing a child. The results further revealed that average standard of living deprivation was 0.000000000403, while average asset index was 0.00000000386. The average food coping index was 0.0000000172.

**Table 2:** Mean distribution of selected demographic variables of urban refugees in Kenya

Variables	Mean	Std. Dev
Age	37.31132	13.04034
Household size	3.5589	2.6814
Gender		
Man	0.5438884	0.4981723
Woman	0.4561116	0.4981723
Country of origin		
Burundi	0.0414614	0.1993958
Congo	0.0024631	0.0495782
Democratic Republic of Congo	0.2877668	0.4528148
Eritrea	0.0205255	0.1418182
Ethiopia	0.0931856	0.290752
Rwanda	0.0192939	0.1375843
Somalia	0.341133	0.4741873
South Sudan	0.1711823	0.3767455
Sudan	0.0024631	0.0495782
Uganda	0.0131363	0.1138817
Other countries	0.0073892	0.0606831
NHIF subscription	0.2489746	0.0087595
Community Based Organization membership	0.0742412	0.0053106
Bank loans access	0.0090238	0.0019156
Community savings loans access	0.0114848	0.0021584
Family, relative, friend loans access	0.3962264	0.0099079
Village savings	0.0098441	0.0019999
Bank savings	0.0274815	0.0033116
MPESA savings	0.5566038	0.0100633
Pillow savings	0.1767842	0.0077277
Separated child in household	0.0344545	0.0036947
Physical disability	0.0328138	0.0036087
Cognitive disability	0.0139459	0.0023754
Sensory disability	0.0246103	0.0031385
Person 60 and above	0.0726005	0.0052562
Pregnant or nursing mother	0.0471698	0.0042945
Serious medical problem	0.0828548	0.0055841
Standard of living index deprivation	-4.03e-10	0.028379
Asset index	3.86e-09	0.0330033
Food coping index	1.72e-08	0.0259496

### Health insurance subscriptions across refugees’ demographic variables

**Fig. 1:**
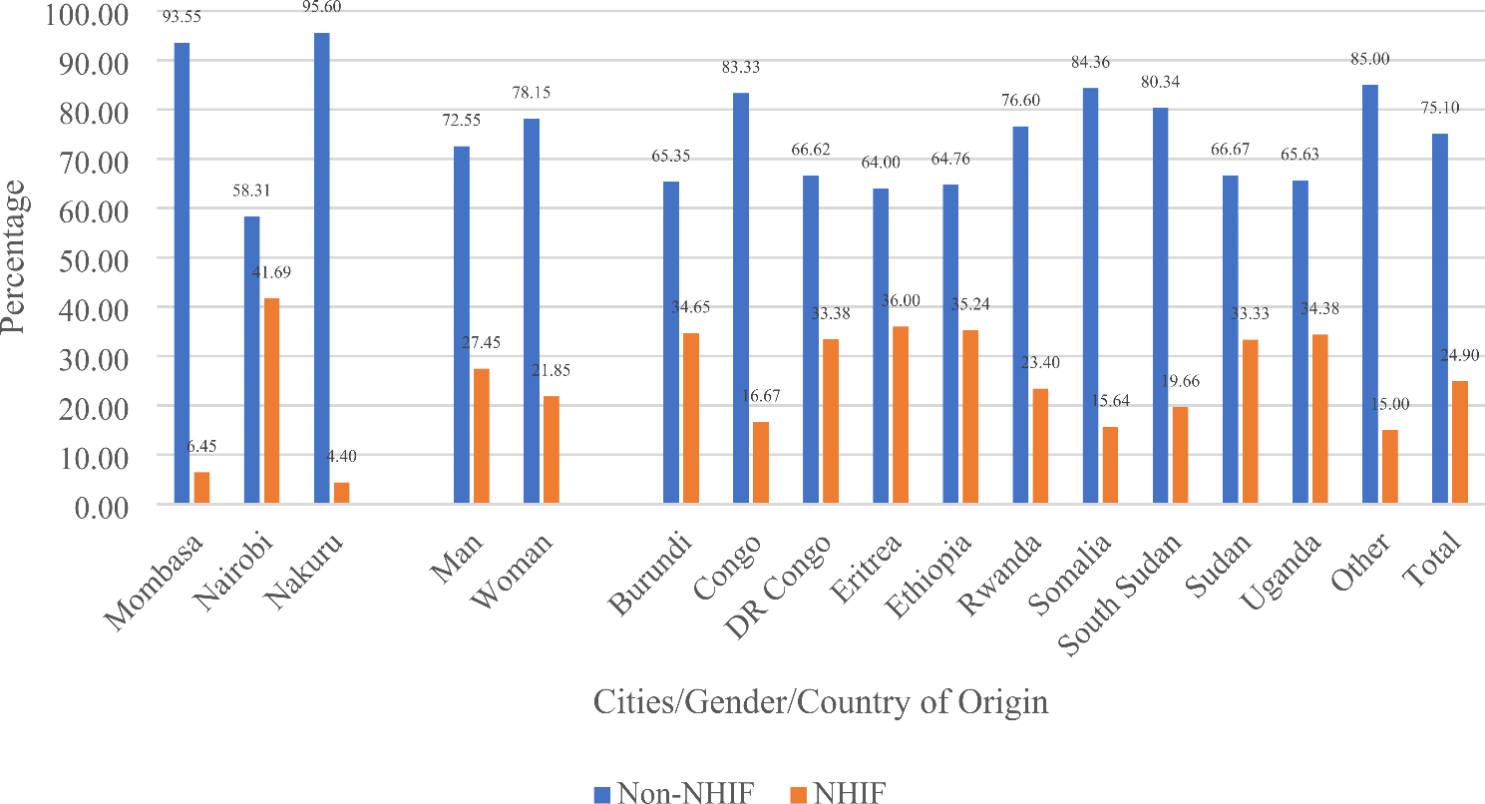
NHIF and Non-NHIF subscribers across Kenya’s urban refugees’ gender, cities and countries of origin

Figure 1 presents the descriptive results of NHIF and Non-NHIF subscribers across the selected Kenyan cities. It reveals that Nairobi had 41.69% NHIF subscribers, followed by Mombasa (6.45%) and Nakuru (4.40%). Figure 1 further shows that across gender, 27.45% and 21.85% of male and female headed households subscribed to NHIF. Across the countries of origin, refugees from Eritrea, Ethiopia and Burundi had highest NHIF subscription rates of 36.00%, 35.24% and 34.65%, respectively. However, those with the lowest subscription rates were refugees from other countries (15.00%), Somalia (15.64%) and Congo (16.67%).

**Fig. 2:**
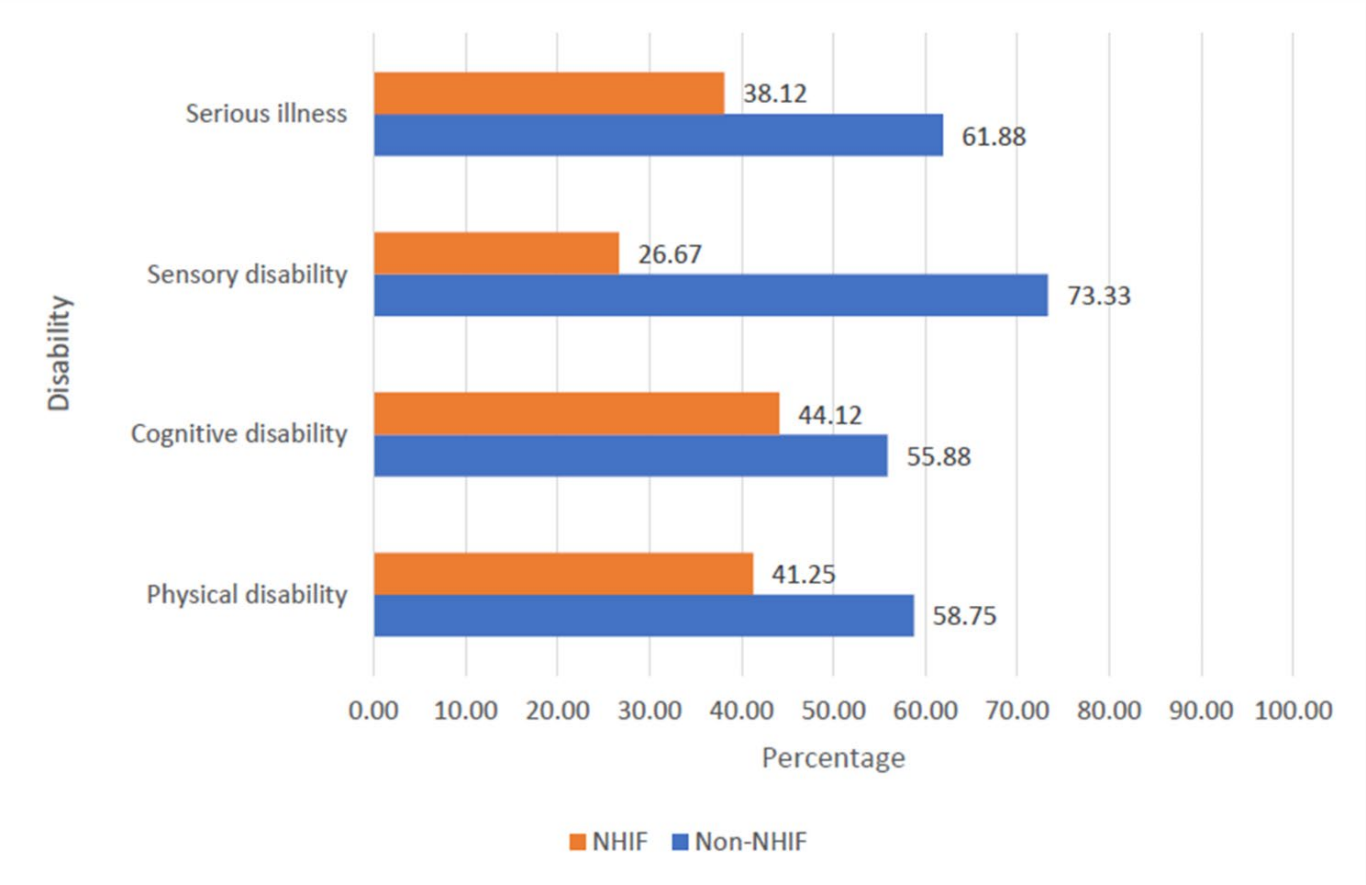
NHIF and Non-NHIF subscribers across disabled/seriously ill respondents

Figure 2 presents the NHIF and Non-NHIF subscribers across reported presence of disability and serious illness. It was revealed that 41.25% of the respondents who had physical disability subscribed to NHIF. Similarly, 44.12% of those with cognitive disability subscribed to NHIF as against 26.67% for those with sensory disability. Among those with serious illness, 38.12% subscribed to NHIF.

**Fig. 3:**
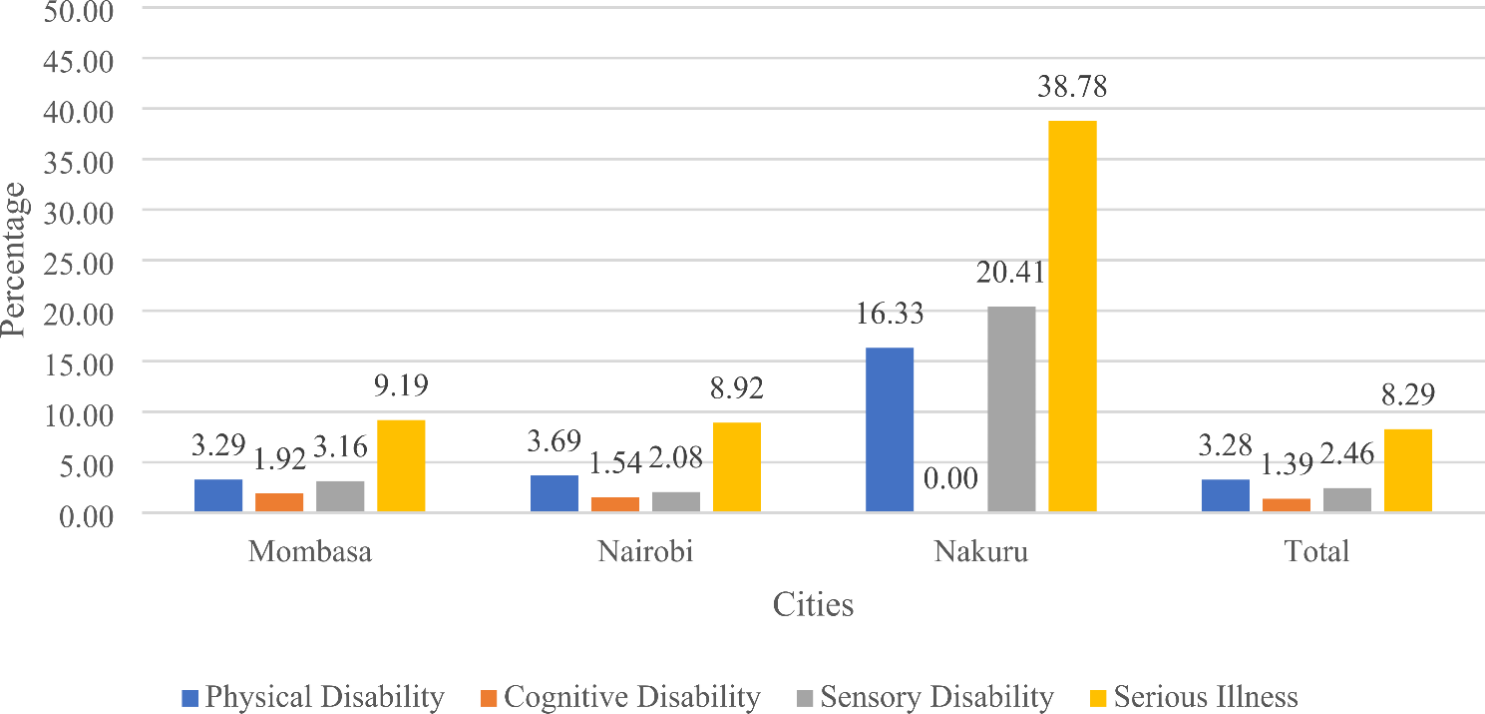
Types of disabilities/serious illness across different cities

Figure 3 shows the different types of disabilities or serious illness across the three cities. The results revealed that across all the cities, some of the households had members who were suffering from serious illness with Nakuru having the highest proportion (38.7%), followed by Mombasa with 9.19% and Nairobi with 8.92%. On the other hand, cognitive disability was least reported across the cities. Nakuru did not have any respondents with cognitive disability/serious illness, Nairobi had 1.54%, while Mombasa had 1.92%. Regarding the households with member(s) suffering from physical disability, Nakuru had the highest number (16.33%), followed by Nairobi with 3.69% and Mombasa with 3.29%. Lastly, it was found that 20.41% of the households who had member(s) with sensory disability was from Nakuru, followed by 3.16% from Mombasa and 2.08% from Nairobi.

### Determinants of NHIF subscription

This section presents the results of the correlates of NHIF subscription among urban refugees in Kenya. Table 3 presents the conventional Probit results of the factors influencing Kenyan urban refugees’ subscription to the National Health Insurance Fund (NHIF). The model produced a good fit for the data as shown by the statistical significance (*p* < 0.01) of the LR Chi-Square statistics. The results showed that membership of CBOs significantly and positively influenced (*p* < 0.01) the probability of subscribing to NHIF. This implies that refugees who were members of CBOs had higher probability of subscribing to NHIF. The results further revealed that at 10% level of statistical significance, Kenyan urban refugees who used informal loans had significantly lower probability of subscribing to NHIF, while those who had bank savings had higher probability of subscription (*p* < 0.01).

Subscription to NHIF was significantly and positively influenced by higher asset index (*p* < 0.05). This implies that an increase in asset index increases the probability of subscribing to NHIF. The results also revealed that subscription to NHIF was significantly and positively influenced by food insecurity coping index (*p* < 0.05). This implies that an increase in urban refugees’ food insecurity coping index increased the probability of subscribing to the NHIF. It is also revealed that compared to Mombasa residents, subscription to NHIF was significantly and positively influenced by residence in Nairobi (*p* < 0.01), while it was negatively influenced by residence in Nakuru (*p* < 0.10). These results imply that compared to refugees who resided in Mombasa, refugees in Nairobi had higher probability of subscribing to NHIF, while those in Nakuru had lower probability. In addition, household size is with positive sign and statistically significant (*p* < 0.01). This implies that as household size increased, the probability of subscribing to NHIF increased.

**Table 3:** Factors influencing the subscription to the National Health Insurance Fund (NHIF)

Variables	Coef.	Std. Err.	Z
Community Based Organization membership	0.4330609***	0.1103811	3.92
Bank loans	− 0.0323272	0.3221376	-0.10
Informal loans	− 0.5809919*	0.3231787	-1.80
Loan from friends /relative loans	− 0.0078217	0.0669364	-0.12
Cooperative savings	− 0.1710995	0.3122306	-0.55
Bank savings	0.4712514***	0.1746202	2.70
Community savings loans access	− 0.0006341	0.0723472	-0.01
Family, relative, friend loans access	0.0880689	0.0885805	0.99
Separated child in household	− 0.1149203	0.1814742	-0.63
Person above 60 in household	− 0.0186304	0.1434964	-0.13
Pregnant woman in household	0.0331572	0.140971	0.24
Standard of living index deprivation	− 0.038656	0.025659	-1.51
Asset index	0.0478512**	0.0209266	2.29
Food coping index	0.0625961**	0.0260233	2.41
Nairobi City	1.349542***	0.0868365	15.54
Nakuru City	− 0.4629731***	0.1465544	-3.16
Household size	0.0912114***	0.0125713	7.26
Constant	-1.92518***	0.1056793	-18.22
Number of obs	2403		
LR chi2(17)	600.55		
Prob > Chi2	0.0000		
Log likelihood = -1044.6621			

Please note: *** 1% level of significance, ** 5% level of significance and *10% level of significance

**Table 4:** Treatment effects parameters for subscription to the National Health Insurance Fund (NHIF)

Indicators/Treatment	Physical disability	Cognitive disability	Sensory disability	Serious illness
Potential outcome mean (treated)	0.3545***	0.4263***	0.3019***	0.3442***
Z statistics	7.19	5.89	8.59	10.16
Potential outcome mean (control)	0.2434***	0.2457***	0.2468***	0.2396***
Z statistics	27.41	27.78	27.77	26.45
Average Treatment Effect (ATE)	0.1100**	0.1816**	0.0533	0.1046***
Z statistics	2.22	2.53	1.54	3.04
Average Treatment Effect on the Treated (ATET)	0.1251**	0.1117	0.0294	0.0996***
Z statistics	2.45	1.42	0.61	3.06

Table 4 presents the parameters of the potential outcome mean, average treatment effect and average treatment effect on the treated across the different forms of disability and presence of serious illness among households’ members. It reveals that the average potential outcome means for the treated groups are statistically significant (*p* < 0.01) with 0.3545, 0.4263, 0.3019 and 0.3442 for households that indicated member(s) suffering from physical disability, cognitive disability, sensory disability and serious illness, respectively. These results can be compared with those for the control group which also showed statistical significance (*p* < 0.01) with 0.2434, 0.2457, 0.2468 and 0.2396 for households that indicated member(s) suffering from physical disability, cognitive disability, sensory disability and serious illness, respectively.

Moreover, the values for ATEs are all positive and statistically significant in the estimates for physical disability, cognitive disability and serious illness (*p* < 0.05). Specifically, the probability of subscribing to NHIF for those with physical disability is significantly higher by 0.1100, when compared with the control group. Similarly, the households that indicated to have members with cognitive disability had their average probability of subscribing to NHIF being significantly higher (*p* < 0.05) by 0.1816. In addition, the households that indicated to have members with serious illness had their average probability of subscribing to NHIF being significantly higher (*p* < 0.01) by 0.1046 when compared with the control group. The results for ATET revealed that households that indicated to have member(s) with physical disability had their average probability of subscribing to NHIF being significantly higher (*p* < 0.05) by 0.1251 compared to if such disability is absent. The ATET results for households that indicated to have members with serious illness revealed that their average probability of subscribing to NHIF was significantly higher (*p* < 0.01) by 0.0996 compared to if they never had member(s) with such illness.

## Discussion

Health insurance is the bedrock of UHC because it ensures timely access to quality healthcare services with less concern on some derogatory financial constraints [54, 55]. Therefore, the goal of health policies in revitalizing the NHIF is to sustain international standard and quality of healthcare infrastructure and services and promote utilization equity. However, it should be noted that the operation mechanisms of the Kenyan NHIF have not guaranteed healthcare service access by many subscribers. This can be substantiated by the finding of Mutai et al. [56] who submitted that access to healthcare services under the NHIF was at 30% in Nairobi County and Makadara Constituency.

The results revealed that 24.90% of all the refugees subscribed to NHIF, and subscriptions were highest in Nairobi (41.69%). The results are in accordance with previous finding, which indicated that 25.3% of the migrant population in Kenya were health insured [44]. It had also been emphasized that 25% of Kenyan host population was health insured [20]. However, the finding is different from that of Quartey et al. [43] who studied some vulnerable stateless people in Ghana and reported 48% health insurance subscription. The difference between health insurance subscription among refugees in Kenya and Ghana can be explained from the high coverage of insurance in Ghana, which was reported by Ayanore [52] as 66% among males and 52.6% among females. We also found a wide variation in health insurance subscription among the cities. This is in line with our expectation. Specifically, being the capital city and the most COVID-19 affected city, refugees in Nairobi may have a higher health insurance subscription rate due to high concentration and accessibility of health infrastructure, service quality, specialization, and high level of employment opportunities.

Another fundamental factor influencing demand for healthcare services is income. Although this was not directly captured in our study due to data limitations, we included asset and housing deprivation indices as proxies for income. Our findings with respect to these variables are in line with expectations since assets and food insecurity coping indices promoted NHIF subscription, while housing deprivation reduced it. The fundamental role of household’s physical asset portfolio on the demand for insurance had been emphasized in some empirical and theoretical literature. A positive correlation between demand for life insurance and net asset and wealth had been emphasized by Hav [57], while insurance demand had been closely linked to asset accumulation and income [58–60]. However, the findings in this study can be compared to those of Tian and Dong [61] who reported a negative insignificant relationship between asset portfolio and subscription to health insurance, although the expected positive and significant associations for incomes and expenditures were found.

The results also revealed that deprivations in basic housing facilities reduced NHIF subscription. Although the literature on the linkage between poverty and health insurance are currently growing with diverse findings, our finding is in line with expectation. Multidimensional housing deprivation reflects the basic household’s standard of living. In some previous studies, insurance was found to reduce multidimensional deprivations [62]. In another related study, out-of-pocket payment was found to induce a long-term multidimensional poverty in Nigeria, India and Columbia [63]. Similarly, multidimensional poverty was found to decline with implementation of long-term care insurance (LTCI) among older and middle-aged adults in China [64]. In another related study, Mohanty et al. [65] found that multidimensional poverty was related to incurring of catastrophic health expenditures.

Access to financial resources is theoretically linked to health insurance subscriptions. This may be in the form of informal or formal savings and utilization of some banking services. We found that possession of bank savings increased the probability of subscribing to NHIF. This is expected because possession of a bank savings account may indicate regular inflow of income or being gainfully employed. This is fundamental during the COVID-19 pandemic because jobs were lost, and many may not be able to subsequently pay their monthly subscription rates for NHIF. Our finding can be related to a study in Cameroon, where access to regular financial means of paying monthly subscription was highlighted as one of the barriers to health insurance subscription [66]. In a very related study, lack of health insurance coverage was found to be associated with being unbanked [67]. Moreover, financial stress demotivates people from deploying significant commitments to health enhancing products like insurance [66]. Our finding also indicates a negative association between informal loans and subscription to NHIF. This is in tandem with theoretical expectation since informal borrowing could signal significant financial stress.

The findings further revealed that membership of CBOs was positively and significantly associated with subscription to the NHIF. Essentially, membership of CBOs is a form of social capital which can promote access to information and other personal financial development assistances for the successful implementation of health insurance schemes [68]. Membership of a CBO can also promote collective decisions for the enhancement of members’ welfare. This is in line with the submission of Woolcock and Narayan [69] who noted that “social capital refers to the norms and networks that enable individuals to act collectively”. In some previous studies, the role of social capital in promoting the importance attached to individuals’ health had been advanced [69–72]. Similar views had been expressed by Kawachi et al. [73], Baum [74], and Hsiao and Cheng [75].

The findings from ATE and ATET also revealed the positive role of disability and serious illness in promoting health insurance subscription among the refugees. These are in accordance with the theoretical proposition of adverse selection, which emphasized information asymmetry that often promotes the tendency of people with some form of health vulnerability to demand for health insurance policy [76–79]. In addition, on serious illness, our finding is in alignment with that of Kibu et al. [66] who submitted that presence of serious illness promotes subscription to community-based health insurance.

## Conclusion

The need for UHC as a primary health goal in the SDG-3 has mandated many countries to revisit their health insurance programmes and coverage. Although the Kenyan government implemented the NHIF several decades ago, the operational mechanisms of the programme for meeting the health needs of subscribers still need to be revisited for efficiency and coverage promotion. More importantly, in the contexts of the COVID-19 pandemic and refugees, who are among the most vulnerable segment of any country’s populations, a clearer understanding of access to healthcare services through health insurance is of significant policy relevance. This study has unfolded the role of disability and serious illness in facilitating subscription to health insurance scheme by refugees in Kenya. Our findings have emphasised the need to promote health insurance schemes among vulnerable households, such as those suffering from some form of disability and serious illness. The results also underscore the role of community-based organization in advancing some positive influences on members’ decisions to be health insured. Therefore, government can utilize the CBOs to promote engagement in some information dissemination platforms on issues related to health insurance. In addition, our findings indirectly relay the role of income – through asset index, housing deprivation index, and food insecurity coping index – on health insurance subscription decision. Therefore, initiatives to promote refugees’ welfare will go a long way in facilitating their decision to subscribe to NHIF. Finally, promotion of planned bank savings among refugees through elimination of documentation barriers and interest rate incentives have some potentials to promote health insurance subscription.

## Electronic supplementary material

Below is the link to the electronic supplementary material.

Supplementary Material 1

## Data Availability

The dataset used for this study is under a restricted domain of the World Bank Microdata Library. We obtained authorization to use the data and do not have any authorization to distribute them. The authorization to access the data can be obtained from the UNHCR and World Bank.

## Abbreviations

ATE: Average Treatment Effect
ATET: Average Treatment Effect on the Treated
CATI: Computer Assisted Telephone Interview
CBOs: Community-Based Organizations
FBOs: Faith Based Organizations
NGOs: Non-Governmental Organizations
NHIF: National Hospital Insurance Fund
PCA: Principal Component Analysis
SDGs: Sustainable Development Goals
UHC: Universal Health Coverage
UNHCR: United Nations High Commission on Refugees
