# Supplementary material for: Effect of households’ members disability and serious illness on public health insurance subscription among urban refugees during the COVID-19 pandemic in Kenya
Source: BMC Public Health. 2024 Nov 26;24:3276. doi: 10.1186/s12889-024-20794-1 (PMC11590370; doi:10.1186/s12889-024-20794-1)
Supplement: Supplementary file 1 — Supplementary Material 1 [file 12889_2024_20794_MOESM1_ESM.pdf]

SOCIOECONOMIC ASSESSMENT (SES) FORM

12/1/2020

GENERAL SETTING

SECTION 1: INTRODUCTION

SOCIOECONOMIC ASSESSMENT SURVEY (SES)

INSTRUCTION:

INSTRUCTIONS AND DEFINITIONS ARE PROVIDED IN BOLD UPPERCASE LETTERS THROUGHOUT THE SURVEY.

QUESTIONS FOR THE PARTICIPANTS ARE PROVIDED IN LOWERCASE LETTERS.

SURVEYORS SHOULD READ THESE NOTES SILENTLY AND CLARIFY IF NECESSARY WITH THE TEAM LEADER OR BENEFICIARY.

UPON REACHING THE TARGET RESPONDENT, REMEMBER FIRST TO ESTABLISH THE IDENTITY OF THE "SES SURVEY HOUSEHOLD".THE IDENTITY OF THE "SES HOUSEHOLD" MAY INCLUDE MULTIPLE "PROGRES FAMILIES".

[enumerator_name] select_one

1.1 ENUMERATOR NAME:

○ Customised choices pulled from external database

[enmerator_name_c] skip logic=[${enumerator_name}!=0] select_one

1.2 PLEASE CONFIRM THAT YOU ARE: ${enumerator_name}

○ Yes [1]

[enumerator_name_s] skip logic=[${enumerator_name}=0] text

1.3 PLEASE SPECIFY YOUR NAME

[enumerator_team] select_one

1.4 PLEASE SELECT YOUR TEAM NUMBER.

○ 1 [1]
○ 2 [2]

[idd_i] skip logic=[${progresdata}=1] select_one

1.5 SELECT THE FIRST 3 DIGITS OF THE PROGRES ID FOR THE FAMILY NO.${position_hh}

○ Customised choices pulled from external database

[d21_i] skip logic=[${progresdataind}=1] select_one

1.6 TO RESET THIS FAMILY, PRESS THE BELOW OPTION FOR A FEW SECONDS, AND THEN SELECT "Remove group" TO RESTART THE SEARCH.

○ PRESS DOWN TO REMOVE [1]

[iddd_i] skip logic=[${progresdata}=1 and ${idd_i}!=0] text

1.7 SEARCH THE PROGRES ID BY ENTERING AT LEAST 3 DIGITS FOR THE FAMILY NO.${position_hh}:

[progres_hhid_i] skip logic=[${progresdata}=1 and ${idd_i}!=0] select_one

1.8 SELECT THE PROGRES ID FOR THE FAMILY NO.${position_hh}:

○ Customised choices pulled from external database

[d2_i] skip logic=[${progresdataind}=1 and ${idd_i}!=0] select_one

1.9 TO RESET THIS FAMILY, PRESS THE BELOW OPTION FOR A FEW SECONDS, AND THEN SELECT "Remove group" TO RESTART THE SEARCH.

○ PRESS DOWN TO REMOVE [1]

[progres_hhid_other_i] skip logic=[${progresdata}=0 or ${idd_i}="0" or ${progres_hhid_i}="0"] text

1.10 PLEASE ENTER THE PROGRES HOUSEHOLD ID

YOU ARE TRYING TO REACH HOUSEHOLD ID: ${show_hhid_i}.THE HEAD OF HOUSEHOLD IS: ${n_progres_i}${sex_progres_i}${age_progres_i} years oldfrom ${coo_progres_i} currently living in ${location_progres_i}.

[members_read_only] select_multiple

1.11 THIS FAMILY INCLUDES THE FOLLOWING MEMBERS:

□ Customised choices pulled from external database

BELOW ARE THE PHONE NUMBERS AVAILABLE TO CALL THIS HOUSEHOLD. TRY EACH PHONE NUMBER (IN LISTED ORDER) TO REACH THIS HOUSEHOLD.TRY TO SPEAK TO THE TARGET RESPONDENT (${n_progres_i}). BUT, IF UNAVAILABLE OR DECEASED, YOU MAY SPEAK TO ANYONE IN THE CURRENT HOUSEHOLD OF ${n_progres_i}.

[phone] select_one

1.12 ONCE REACHED, SELECT THE PHONE NUMBER THAT REACHED THIS HOUSEHOLD BEFORE CONTINUING TO THE NEXT PAGE.

○ Customised choices pulled from external database

[phone_other] skip logic=[${phone}=0] integer

1.13 ENTER OTHER PHONE NUMBER

INTRODUCE YOURSELF: Hello, my name is ${enumerator_name}. I am calling on behalf of the United Nations High Commissioner for Refugees (UNHCR) and the World Bank. The World Bank and UNHCR are trying to help assess the livelihoods of refugees in urban areas. Your household has been randomly chosen to participate in this survey. Your cooperation and answers would be extremely important. All personal information provided will be kept strictly confidential.

[speaking_whom] select_one

1.14 WHO ARE YOU SPEAKING WITH?

- ○ ${n_progres_i} [1]
- ○ ${n_progres_i} unavailable, other member of the target household [2]
- ○ ${n_progres_i} deceased, other member of the target household [3]
- ○ I am not 100% sure, but probably from the target household [4]
- ○ Someone outside the target household [5]

[why_not_sure] skip logic=[${speaking_whom}=4] text

1.15 WHY ARE YOU NOT SURE?

[age_confirmation] skip logic=[${speaking_whom}=1 or ${speaking_whom}=2 or ${speaking_whon select_one

1.16 Are you 15 years or older?

- ○ Yes [1]
- ○ No [0]

[age_confirmation2] skip logic=[${age_confirmation}=0] select_one

1.17 Is there anyone else living in the household that is older than 15 years old?

- ○ Yes [1]
- ○ No [0]

[age_confirmation3] skip logic=[${age_confirmation2}=1] select_one

1.18 May I speak to that person now?

- ○ Yes [1]
- ○ No [0]

GO BACK TO THE QUESTION 1.14: "WHO ARE YOU SPEAKING WITH?" TO RESELECT.

[age_confirmation4] skip logic=[${age_confirmation3}=0] select_one

1.19 Can I reach that person on a different phone number?

- ○ Yes [1]
- ○ No [0]

[another_phone] skip logic=[${age_confirmation4}=1] text

1.20 ENTER ANOTHER PHONE NUMBER:

[another_day] skip logic=[${age_confirmation4}=0] select_one

1.21 Can I call again another day?

- ○ Yes [1]
- ○ No [0]

[someone_outside] select_one

1.23 TRY TO ASCERTAIN WHETHER THIS PERSON KNOWS ANY MEMBERS OF THE TARGET proGres HOUSEHOLD.IF THEY DO, ASK WHETHER THEY CAN HELP YOU REACH ANYONE IN THE HOUSEHOLD OF THE TARGET RESPONDENT, AND GIVE YOU THEIR PHONE NUMBER. (BELOW YOU CAN SEE THE NAMES OF THE HOUSEHOLD MEMBERS).

○ Respondent does not know any member of the target household [1]
○ Respondent knows the target household, but does not have a phone number [2]
○ Respondent knows a phone number for the target respondent's household [3]
○ Repondent knows the target household, and can hand over the phone to the member of the household [4]

[members_read_only2] select_multiple

1.24 THIS FAMILY INCLUDES THE FOLLOWING MEMBERS:

□ Customised choices pulled from external database

[outsider_phone] skip logic=[${someone_outside}=3] integer

1.25 ENTER THE PHONE NUMBER:

[currently_reside] skip logic=[${age_confirmation}=1 or ${someone_outside}=4] select_one

1.26 Where do you and the members of your household currently reside in Kenya?

○ Mombasa [1]
○ Nakuru [2]
○ Nairobi [3]
○ Kalobeyei settlement [4]
○ Kakuma camp [5]
○ Dadaab camp [6]
○ Other area in Kenya [99]
○ Other country [100]

[county_code_before] skip logic=[${currently_reside}=99] select_one

1.27 Please specify county

○ Mombasa [1]
○ Kwale [2]
○ Kilifi [3]
○ TaRiver [4]
○ Lamu [5]
○ Taita/Taveta [6]
○ Garissa [7]
○ Wajir [8]
○ Mandera [9]
○ Marsabit [10]
○ Isiolo [11]
○ Meru [12]
○ Tharaka-Nithi [13]
○ Embu [14]
○ Kitui [15]
○ Machakos [16]
○ Makueni [17]
○ Nyandarua [18]
○ Nyeri [19]
○ Kirinyaga [20]
○ Murang'a [21]
○ Kiambu [22]
○ Turkana [23]
○ WePokot [24]
○ Samburu [25]
○ TraNzoia [26]
○ UasGishu [27]
○ Elgeyo/Marakwet [28]
○ Nandi [29]
○ Baringo [30]
○ Laikipia [31]
○ Nakuru [32]
○ Narok [33]
○ Kajiado [34]
○ Kericho [35]
○ Bomet [36]
○ Kakamega [37]
○ Vihiga [38]
○ Bungoma [39]
○ Busia [40]
○ Siaya [41]
○ Kisumu [42]
○ HoBay [43]
○ Migori [44]
○ Kisii [45]
○ Nyamira [46]
○ Nairobi City [47]

[country_code_before] skip logic=[${currently_reside}=100] select_one

1.28 Please specify country

○ Burundi [1]
○ Congo (Brazzaville) [2]
○ Congo, (Kinshasa) [3]
○ Ethiopia [4]
○ Kenya [5]
○ Rwanda [6]
○ Somalia [7]
○ South Sudan [8]
○ Sudan [9]
○ Uganda [10]
○ Other (specify) [99]

[country_code_before_sp] skip logic=[${country_code_before}=99] text

1.29 Please specify other country:

[move_dueto_covid] skip logic=[${age_confirmation}=1 or ${someone_outside}=4] select_one

1.30 Did your household have to move to a new place of residence due to COVID-19 restrictions?

○ Yes [1]
○ No [0]

[where_resided_before] skip logic=[${move_dueto_covid}=1] select_one

1.31 Where did you reside before moving due to COVI-19?

○ Mombasa [1]
○ Nakuru [2]
○ Nairobi [3]
○ Kalobeyei settlement [4]
○ Kakuma camp [5]
○ Dadaab camp [6]
○ Other area in Kenya [99]
○ Other country [100]

[county_code_before2] skip logic=[${where_resided_before}=99] select_one

1.32 Please specify county

○ Mombasa [1]
○ Kwale [2]
○ Kilifi [3]
○ TaRiver [4]
○ Lamu [5]
○ Taita/Taveta [6]
○ Garissa [7]
○ Wajir [8]
○ Mandera [9]
○ Marsabit [10]
○ Isiolo [11]
○ Meru [12]
○ Tharaka-Nithi [13]
○ Embu [14]
○ Kitui [15]
○ Machakos [16]
○ Makueni [17]
○ Nyandarua [18]
○ Nyeri [19]
○ Kirinyaga [20]
○ Murang'a [21]
○ Kiambu [22]
○ Turkana [23]
○ WePokot [24]
○ Samburu [25]
○ TraNzoia [26]
○ UasGishu [27]
○ Elgeyo/Marakwet [28]
○ Nandi [29]
○ Baringo [30]
○ Laikipia [31]
○ Nakuru [32]
○ Narok [33]
○ Kajiado [34]
○ Kericho [35]
○ Bomet [36]
○ Kakamega [37]
○ Vihiga [38]
○ Bungoma [39]
○ Busia [40]
○ Siaya [41]
○ Kisumu [42]
○ HoBay [43]
○ Migori [44]
○ Kisii [45]
○ Nyamira [46]
○ Nairobi City [47]

[country_code_before2] skip logic=[${where_resided_before}=100] select_one

1.33 Please specify country

○ Burundi [1]
○ Congo (Brazzaville) [2]
○ Congo, (Kinshasa) [3]
○ Ethiopia [4]
○ Kenya [5]
○ Rwanda [6]
○ Somalia [7]
○ South Sudan [8]
○ Sudan [9]
○ Uganda [10]
○ Other (specify) [99]

[country_code_before_sp2] skip logic=[${country_code_before}=99] text

1.34 Please specify other country:

CONSENT

VOLUNTARY INFORMED CONSENT FORM

READ THE FOLLOWING TO THE RESPONDENT:

As I was telling you, the World Bank and UNHCR are conducting a phone survey for refugees in Kenya. The objectives of the survey are to better understand refugee's living conditions here in Kenya to help design appropriate policy responses. If you agree to participate in this survey, we will ask you some questions related to housing conditions, employment, education, food security, and coping strategies. The interview will take about 30 minutes, and we will schedule it when it is convenient for you. Any responses that you give us will be confidential, that is, the researchers will not let anyone else know how you answered. The anonymized responses will be used for research purposes and published in reports. We can assure you that we will never include any names or other personal details in publications. The study is voluntary and if you do not want to participate, you can decide that at any time. There will be no negative impacts on you. If at any time you want to stop participating, please let me know and we will respect your decision.No incentives or benefits will be given for participating. However, your answers will help understand the livelihoods and living conditions of refugees in the urban areas.In order to participate, you must be the head of your household (the person that takes key decisions) or should be able to answer questions about your household members, for example level of education, occupation etc.ASK THE RESPONTENT IF SHE/HE HAS ANY QUESTION, AND IF SHE/HE DOES, RESPOND. INFORM THAT HE/SHE CAN ALWAYS CONTACT helpline.kenya@unhcr.org FOR FURTHER QUESTIONS.

[consent] select_one

1.35 Do you agree to participate in the survey?

○ Yes [1]
○ No [0]

[reason_not] skip logic=[${consent}=0] select_one

1.36 What is the main reason for not participating in the survey?

○ I am busy [1]
○ Unable to survey - parent or contact refusal [2]
○ Unable to survey - spouse refusal [3]
○ Unable to survey - in prison [4]
○ Unable to survey - mental illness / disability [5]
○ Unable to survey - other [6]

[reason_not_other] skip logic=[${reason_not}=6] text

1.37 SPECIFY OTHER:

[willing_later] skip logic=[${reason_not}=1] select_one

1.38 Are you willing to schedule the interview for a later date or time?

○ Yes [1]
○ No [0]

RESCHEDULING INSTRUCTIONS: ASK WHEN THEY ARE NEXT AVAILABLE. IF YOU CANNOT PERSONALLY CALL BACK AT THIS TIME, MAKE A TENTATIVE APPOINTMENT. THEN, LET THE RESPONDENT KNOW THAT YOU WILL CONTACT THEM TO CONFIRM WHEN YOU WILL CALL BACK. COMMUNICATE THE TIME WITH YOUR SUPERVISOR TO CONFIRM IF ANOTHER PERSON CAN CALL BACK. RECORD THIS INFORMATION AND THE CURRENT TIME ON THE TRACKING SHEET NOW. END THE INTERVIEW. DO NOT SUBMIT THIS FORM. ONLY SUBMIT A FORM IF YOU EITHER REACHED A HOUSEHOLD, OR EXHAUSTED ALL ATTEMPTS.

READ THE FOLLOWING TO THE RESPONDENT:

Thank you very much for your time. If you change your mind and would like to participate in the interview, you may call me back any time. Here is my phone number - MENTION YOUR PHONE NUMBER.

[reason_refuse] skip logic=[${consent}=0] select_multiple

1.39 RECORD YOUR IMPRESSIONS OF WHY THE RESPONDENT REFUSES TO PARTICIPATE. IF YOU FEEL COMFORTABLE DOING SO, YOU MAY ASK WHY: Why don't you want to participate in this survey round? Choose up to 3 reasons.

□ Survey is too long [1]
□ has caregiving duties [2]
□ has to work [3]
□ does not want to disclose personal information [4]
□ is suspicious of REMIT/KNBS/WB [5]
□ just doesn't want to / no reason given [6]
□ Other (specify) [7]

[reason_refuse_other] skip logic=[${reason_refuse}=7] text

1.40 SPECIFY OTHER:

SECTION 2: HH ROSTER

SECTION 2: HH ROSTER

THIS SECTION WILL ASK YOU TO REGISTER THE FAMILY IDENTIFICATION NUMBER(S), AND ASK BASIC INFORMATION ABOUT THE FAMILY(S) AND ITS MEMBERS.

[family_number] skip logic=[${multiplefamily}=1] integer

2.1 HOW MANY PROGRES FAMILIES ARE IN THIS HOUSEHOLD?

REPEAT STARTS: HOUSEHOLD OF (${progres_hhid_i})

ADD A FAMILY ${position_hh}

NOTE:

PLEASE MAKE SURE TO FIRST REGISTER THE FAMILY OF THE TARGET RESPONDENT.

[idd] skip logic=[${progresdata}=1] select_one

2.2 SELECT THE FIRST 3 DIGITS OF THE PROGRES ID FOR THE FAMILY NO.${position_hh}

○ Customised choices pulled from external database

[d21] skip logic=[${progresdataind}=1 and ${oncehhid}!=""] select_one

2.3 TO RESET THIS FAMILY, PRESS THE BELOW OPTION FOR A FEW SECONDS, AND THEN SELECT "Remove group" TO RESTART THE SEARCH.

○ PRESS DOWN TO REMOVE [1]

[iddd] skip logic=[${progresdata}=1 and ${idd}!=0] text

2.4 SEARCH THE PROGRES ID BY ENTERING AT LEAST 3 DIGITS FOR THE FAMILY NO.${position_hh}:

[progres_hhid] skip logic=[${progresdata}=1 and ${idd}!=0] select_one

2.5 SELECT THE PROGRES ID FOR THE FAMILY NO.${position_hh}:

○ Customised choices pulled from external database

[d2] skip logic=[${progresdataind}=1 and ${idd}!=0 and ${oncehhid}!=""] select_one

2.6 TO RESET THIS FAMILY, PRESS THE BELOW OPTION FOR A FEW SECONDS, AND THEN SELECT "Remove group" TO RESTART THE SEARCH.

○ PRESS DOWN TO REMOVE [1]

[progres_hhid_other] skip logic=[${progresdata}=0 or ${idd}="0" or ${progres_hhid}="0"] text

2.7 PLEASE ENTER THE PROGRES HOUSEHOLD ID

THE FOLLOWING INFORMATION ARE FOUND IN THE REGISTRATION DATA FOR THIS FAMILY (${show_hhid}).

- The family size (active only) is ${fs_progres}
- The Principal Applicant is ${n_progres}

[IDDDD2] skip logic=[${progresdataind}=1] select_multiple

2.8 IF THIS IS CORRECT, PLEASE CONFIRM THE INDIVIDUAL MEMBERS IN THIS FAMILY (${show_hhid}) :

□ Customised choices pulled from external database

[headhh1] skip logic=[${progresdataind}=1] select_one

2.9 SELECT THE HEAD OF THIS FAMILY :

○ Customised choices pulled from external database

[respondant1] skip logic=[${progresdataind}=1 and ${position_hh}=1] select_one

2.10 SELECT THE MAIN RESPONDENT OF THIS SURVEY :

○ Customised choices pulled from external database

IF THIS INFORMATION IS CORRECT, PROCEED TO THE NEXT.

[d22] select_one

2.11 IF NOT, RESET THIS FAMILY BY PRESSING THE BELOW OPTION FOR A FEW SECONDS, AND THEN SELECT "Remove group" TO RESTART THE SEARCH.

○ PRESS DOWN TO REMOVE [1]

[IDDDD2_add] skip logic=[selected(${IDDDD2},'0') or ${progresdataind}=0 or ${idd}="0" or ${pr integer

2.12 HOW MANY MEMBERS DO YOU NEED TO ADD FOR THIS FAMILY (${show_hhid})?

REPEAT STARTS: MEMBER

ADD A MEMBER ${position_m}

NOTE:

PLEASE MAKE SURE TO FIRST REGISTER THE HEAD OF THIS FAMILY.

[name2] text

2.13 THE NAME OF ${text_hhh}:

[gender2] select_one

2.14 SEX:

○ Male [1]
○ Female [2]

[age2] integer

2.15 AGE:

[refugee2] select_one

2.16 IS HE/SHE A REFUGEE?

○ Yes [1]
○ No [0]

[country_origin2] select_one

2.17 COUNTRY OF ORIGIN:

○ Customised choices pulled from external database

[relationship2] skip logic=[${position_m}>1] select_one

2.18 RELATIONSHIP TO THE HEAD OF FAMILY:

○ Spouse [1]
○ Son / Daughter [2]
○ Step-Son / Step-Daughter [3]
○ Brother / Sister [4]
○ Step-Brother / Step-Sister [5]
○ Father / Mother [6]
○ Step-Father / Step-Mother [7]
○ Grandparent [8]
○ Grandchild [9]
○ Uncle / Aunt [10]
○ Nephew / Niece [11]
○ In-Law [12]
○ Other relative [-96]
○ Non-Relative [13]

[head2] skip logic=[(${progresdataind}=0 or ${idd}="0" or ${progres_hhid}="0") and ${pc select_one

2.19 IS THIS THE HEAD OF THIS FAMILY?

○ Yes [1]

[respondant2] skip logic=[${oncehhid}="" and ${position_hh}=1 and (${progresdataind}=0 or (select_one

2.20 IS THIS YOUR MAIN RESPONDENT?

○ Yes [1]
○ No [0]

[IndividualID_seperate] text

2.21 (OPTIONAL): IF HE/SHE HAS A PROGRES INDIVIDUAL ID NUMBER, PLEASE NOTE THIS.

[d3] select_one

2.22 IN CASE YOU NEED TO RESET THIS MEMBER, PRESS THE BELOW OPTION FOR A FEW SECONDS, AND THEN SELECT "Remove group".

○ PRESS DOWN TO REMOVE [1]

REPEAT ENDS: MEMBER

[w1] skip logic=[${position_hh}=1 and sum(${agecheck1})=1] select_one

2.23 THE RESPONDENT'S AGE MUST BE OVER 15. PLEASE REVIEW.

○ Warning [1]

[w2] skip logic=[(${oncehhid} and ${show_hhid}!=${oncehhid}) or ${oncename2}!="" select_one

2.24 YOU HAVE POSSIBLY MIXED TWO FAMILIES. THIS HAPPENS WHEN YOU HAVE SELECTED FAMILY MEMBERS, AND RE-SELECT ANOTHER FAMILY. THIS MAY MIXED THE MEMBERS OF TWO FAMILIES.TO RESOLVE THIS, YOU MUST RESET THIS FAMILY AND RE-ENTER.

○ Warning [1]

REPEAT ENDS: FAMILY

[HHHH2] select_one

2.25 AMONG THE HEADS OF FAMILIES, WHO IS THE HEAD OF ALL THE FAMILIES IN THIS HOUSEHOLD?

○ ${h1} [1]
○ ${h2} [2]
○ ${h3} [3]
○ ${h4} [4]
○ ${h5} [5]

PLEASE CONFIRM THAT ALL THE FAMILY(IES) AND FAMILY MEMBERS ARE CORRECTLY REGISTERED.

Family 1 (${family1id}): ${familysize1} member(s)
Family 2 (${family2id}): ${familysize2} member(s)
Family 3 (${family3id}): ${familysize3} member(s)
Family 4 (${family4id}): ${familysize4} member(s)
Family 5 (${family5id}): ${familysize5} member(s)

[w3] skip logic=[(${progresdataind}=0 or indexed-repeat(${idd},${FAMILY},1)="0" or select_one

2.26 THERE ARE ${sumcountr} RESPONDANT(S). PLEASE REVIEW.

○ Warning [1]

[confirmation] skip logic=[not((${progresdataind}=0 or indexed-repeat(${idd},${FAMILY},1)="0 select_one

2.27 CONFIRM THAT THIS INFORMATION IS CORRECT.

○ Yes [1]

[received_call] select_one

2.28 Do you know if any member of your household has received a phone call to complete an interview like this one?

○ Yes [1]
○ No [0]

[id_received_call] skip logic=[${received_call}=1] select_one

2.29 Which (and whose) proGres family is it?

○ ${hh1} [1]
○ ${hh2} [2]
○ ${hh3} [3]
○ ${hh4} [4]
○ ${hh5} [5]

SECTION 3: EDUCATION

SECTION 3: EDUCATION

THIS SECTION INCLUDES QUESTIONS RELATED TO THE EDUCATION LEVEL OF ALL INDIVIDUAL FAMILY MEMBERS AGE 3 YEARS AND GREATER.

READ THE FOLLOWING TO THE RESPONDENT: Now I will ask you about the level of education of all individual family members whose age is above 3 years.

REPEAT STARTS: FAMILY

REPEAT STARTS: MEMBER

START THE QUESTIONS ABOUT:

${ed_name}

[ed_everyn] select_one

3.1 Has ${ed_name} ever attended school (in Kenya or another country)?

○ Yes [1]
○ No [0]

[ed_currentyn] skip logic=[${ed_everyn}=1] select_one

3.2 Is ${ed_name} currently enrolled at school or in another academic institution?

○ Yes [1]
○ No [0]

[ed_covid] skip logic=[${ed_currentyn}=0] select_one

3.3 Was ${ed_name} enrolled at school or in another academic institution before March 20 when the COVID-19 restrictions were enforced?

○ Yes [1]
○ No [0]

[ed_covid2] skip logic=[${ed_covid}=1] select_one

3.4 Were COVID-19 related school measures the main reason for ${ed_name} to stop attending school or, were there other reasons?

○ Yes, the COVID-19 restrictions were the main reasons [1]
○ No, he/she was going to stop attending anyway [0]

[ed_reason_stop] skip logic=[${ed_covid2}=0] select_multiple

3.5 Why did ${ed_name} stop attending?

□ Completed school [1]
□ School cost [2]
□ School is too far from home [3]
□ No female teachers [4]
□ No male teachers [5]
□ School conflicts with beliefs [6]
□ Poor quality of school [7]
□ Lack of transportation [8]
□ Lack of separate toilets for boys and girls [9]
□ Insecurity [10]
□ Own Illness [11]
□ Own disability [12]
□ Family Illness [13]
□ Family disability [14]
□ Not interested anymore [15]
□ Needs to work to support household expenses [16]
□ Needs to do housework (cook, clean, take care of dependents) [17]
□ Parents do not allow [18]
□ Orphaned [19]
□ Lack of financial support [20]
□ Lack of sanitary towels [21]
□ Marriage [22]
□ Pregnancy [23]
□ Other (specify) → SKIP to d [99]
□ Don't know [-98]
□ Refused to respond [-99]

[ed_reason_stop_sp] skip logic=[${ed_reason_stop}=99] text

3.6 PLEASE SPECIFY OTHER REASON:

[ed_reopen] skip logic=[${ed_covid}=0 or ${ed_covid2}=1] select_one

3.7 Will ${ed_name} be enrolled in school when schools are re-opened?

○ Yes [1]
○ No [0]

[ed_reason_not_enrolled] skip logic=[${ed_reopen}=0] select_multiple

3.8 Why will ${ed_name} not be enrolled?

□ Completed school [1]
□ School cost [2]
□ School is too far from home [3]
□ No female teachers [4]
□ No male teachers [5]
□ School conflicts with beliefs [6]
□ Poor quality of school [7]
□ Insecurity [8]
□ Own Illness [9]
□ Own disability [10]
□ Family Illness [11]
□ Family disability [12]
□ Not interested anymore [13]
□ Needs to work to support household expenses [14]
□ Needs to do housework (cook, clean, take care of dependents) [15]
□ Lack of sanitary towels [16]
□ Marriage [17]
□ Pregnancy [18]
□ Other (specify) [99]
□ Don't know [-98]

[ed_reason_not_enrolled_sp] skip logic=[${ed_reason_not_enrolled}=99] text

3.9 PLEASE SPECIFY OTHER REASON:

[ed_currentg] skip logic=[${ed_currentyn}=1 or ${ed_reopen}=1] select_one

3.10 Which grade is or was ${ed_name} currently enrolled in?

○ Baby Class /Kindergarten 1 [1]
○ Nursery/Kindergarten 2 [2]
○ Pre Unit/Kindergarten 3 [3]
○ Standard/Grade 1 [4]
○ Standard/Grade 2 [5]
○ Standard/Grade 3 [6]
○ Standard/Grade 4 [7]
○ Standard/Grade 5 [8]
○ Standard/Grade 6 [9]
○ Standard/Grade 7 [10]
○ Standard/Grade 8 [11]
○ Form 1/Grade 9 [12]
○ Form 2//Grade 10 [13]
○ Form 3/Grade 11 [14]
○ Form 4/Grade 12 [15]
○ Form 5 [16]
○ Form 6 [17]
○ Diploma/Certificate Year 1 [18]
○ Diploma/Certificate Year 2 [19]
○ Diploma Year 3 [20]
○ Higher National Diploma [21]
○ Undergraduate Year 1 [22]
○ Undergraduate Year 2 [23]
○ Undergraduate Year 3 [24]
○ Undergraduate Year 4 [25]
○ Undergraduate Year 5 [26]
○ Undergraduate Year 6 [27]
○ Masters Year 1 [28]
○ Masters Year 2 [29]
○ Phd Year 1 [30]
○ Phd Year 2 [31]
○ Phd Year 3 [32]
○ Adult Basic Education [33]
○ Adult Secondary Education [34]
○ Vocational Training Year 1 [35]
○ Vocational Training Year 2 [36]
○ Madrassa/Duksis [37]
○ Other (specify) [-96]
○ Don’T Know [-98]

[ed_currentg_sp] skip logic=[selected(${ed_currentg}, '-96') and ${ed_everyn}=1] text

3.11 Please specify the grade that ${ed_name} is or was enrolled in:

[ed_current_type] skip logic=[${ed_currentyn}=1 or ${ed_reopen}=1] select_one

3.12 What type of school or institution is ${ed_name} currently attending?

○ Public [1]
○ Private [2]
○ Non-formal [3]
○ Other (Please specify) [-96]
○ Don't know [-98]

[ed_current_type_sp] skip logic=[selected(${ed_current_type}, '-96')] text

3.13 Please specify the type of school or institution that ${ed_name} is currently attending:

[ed_highestlvl] skip logic=[${ed_everyn}=1] select_one

3.14 What is the highest educational grade ${ed_name} has completed?

- ○ Baby Class /Kindergarten 1 [1]
- ○ Nursery/Kindergarten 2 [2]
- ○ Pre Unit/Kindergarten 3 [3]
- ○ Standard/Grade 1 [4]
- ○ Standard/Grade 2 [5]
- ○ Standard/Grade 3 [6]
- ○ Standard/Grade 4 [7]
- ○ Standard/Grade 5 [8]
- ○ Standard/Grade 6 [9]
- ○ Standard/Grade 7 [10]
- ○ Standard/Grade 8 [11]
- ○ Form 1/Grade 9 [12]
- ○ Form 2//Grade 10 [13]
- ○ Form 3/Grade 11 [14]
- ○ Form 4/Grade 12 [15]
- ○ Form 5 [16]
- ○ Form 6 [17]
- ○ Diploma/Certificate Year 1 [18]
- ○ Diploma/Certificate Year 2 [19]
- ○ Diploma Year 3 [20]
- ○ Higher National Diploma [21]
- ○ Undergraduate Year 1 [22]
- ○ Undergraduate Year 2 [23]
- ○ Undergraduate Year 3 [24]
- ○ Undergraduate Year 4 [25]
- ○ Undergraduate Year 5 [26]
- ○ Undergraduate Year 6 [27]
- ○ Masters Year 1 [28]
- ○ Masters Year 2 [29]
- ○ Phd Year 1 [30]
- ○ Phd Year 2 [31]
- ○ Phd Year 3 [32]
- ○ Adult Basic Education [33]
- ○ Adult Secondary Education [34]
- ○ Vocational Training Year 1 [35]
- ○ Vocational Training Year 2 [36]
- ○ Madrassa/Duksis [37]
- ○ Other (specify) [-96]
- ○ Don’T Know [-98]

[ed_highestlvl_sp] skip logic=[selected(${ed_highestlvl}, '-96') and ${ed_everyn}=1] text

3.15 Please specify the highest education grade ${ed_name} has completed:

[ed_stop] skip logic=[${ed_everyn}=0] select_multiple

3.16 Why did ${ed_name} never attend school?

- ◻ School cost [1]
- ◻ Too young to attend school [2]
- ◻ Too old to attend school [3]
- ◻ School is too far from home [4]
- ◻ No female teachers [5]
- ◻ No male teachers [6]
- ◻ School conflicts with beliefs [7]
- ◻ School did not admit [8]
- ◻ Poor quality of school [9]
- ◻ Lack of transportation [10]
- ◻ Lack of separate toilets for boys and girls [11]
- ◻ Social or religious pressure (to not go to school) [12]
- ◻ Insecurity [13]
- ◻ Own Illness [14]
- ◻ Own disability [15]
- ◻ Family Illness [17]
- ◻ Family disability [18]
- ◻ Not interested anymore [19]
- ◻ Needs to work to support household expenses [20]
- ◻ Needs to do housework (cook, clean, take care of dependents) [21]
- ◻ Parents did not allow [22]
- ◻ Orphaned [23]
- ◻ Lack of financial support [24]
- ◻ Lack of sanitary towels [25]
- ◻ Marriage [26]
- ◻ Pregnancy [27]
- ◻ Other (specify) [-96]
- ◻ Don't know [-98]

[ed_stop_sp] skip logic=[selected(${ed_stop}, '-96')] text

3.17 Please specify why ${ed_name} has stoped or has never attended school?

[ed_covid3] skip logic=[${ed_currentyn}=0 and ${ed_covid}=0] select_multiple

3.18 Why was ${ed_name} not enrolled in school before March 20 when the COVID-19 restrictions were enforced?

□ Completed school [1]
□ School cost [2]
□ School is too far from home [3]
□ No female teachers [4]
□ No male teachers [5]
□ School conflicts with beliefs [6]
□ Poor quality of school [7]
□ Lack of transportation [8]
□ Lack of separate toilets for boys and girls [9]
□ Insecurity [10]
□ Own Illness [11]
□ Own disability [12]
□ Family Illness [13]
□ Family disability [14]
□ Not interested anymore [15]
□ Needs to work to support household expenses [16]
□ Needs to do housework (cook, clean, take care of dependents) [17]
□ Parents do not allow [18]
□ Orphaned [19]
□ Lack of financial support [20]
□ Lack of sanitary towels [21]
□ Marriage [22]
□ Pregnancy [23]
□ Other (specify) → SKIP to d [99]
□ Don't know [-98]
□ Refused to respond [-99]

[ed_speak] select_multiple

3.19 Can ${ed_name} speak in English, Swahili, Somali, French, Arabic or another language?

□ Swahili [1]
□ Somali [2]
□ English [3]
□ French [5]
□ Arabic [6]
□ Other [4]
□ No [0]

[ed_speak_sp] skip logic=[selected(${ed_speak},"4")] text

3.20 Please specify which language:

[ed_read] select_multiple

3.21 Can ${ed_name} read in any of these languages?

□ Swahili [1]
□ Somali [2]
□ English [3]
□ French [5]
□ Arabic [6]
□ Other [4]
□ No [0]

[ed_read_sp] skip logic=[selected(${ed_read},"4")] text

3.22 Please specify which language:

[ed_write] skip logic=[${ed_read}!=0] select_multiple

3.23 Can ${ed_name} write in any of these languages?

□ Swahili [1]
□ Somali [2]
□ English [3]
□ French [5]
□ Arabic [6]
□ Other [4]
□ No [0]

[ed_write_sp] skip logic=[selected(${ed_write},"4")] text

3.24 Please specify which language:

REPEAT ENDS: MEMBER

REPEAT ENDS: FAMILY

SECTION 4: EMPLOYMENT

L

SECTION 4: EMPLOYMENT

THIS SECTION INCLUDES QUESTIONS RELATED TO LABOR ACTIVITIES OF ALL HOUSEHOLD MEMBERS WHOSE AGE IS GREATER THAN 5 YEARS.

REPEAT STARTS: HOUSEHOLD

REPEAT STARTS: MEMBER

START THE QUESTIONS ABOUT:${em_name}

[ls_work_employee] select_one

4.1 In the last 7 days, has ${em_name} worked at least one hour as an employee for wage, salary, commission, or any payment in kind, including doing paid domestic work or farm work ?

○ Yes [1]
○ No [0]

[ls_work_se] select_one

4.2 In the last 7 days, has ${em_name} worked at least one hour on their own account or as an employer in a non-farm business enterprise?

○ Yes [1]
○ No [0]

[ls_work_seag] select_one

4.3 In the last 7 days, has ${em_name} worked at least one hour on their own or family account or as an employer on a farm holding owned or rented, whether in cultivating crops or in other farm maintenance tasks, or have they cared for livestock ?

○ Yes [1]
○ No [0]

[ls_work_help_nonfarm] select_one

4.4 In the last 7 days, has ${em_name} helped (for at least one hour) in a non-farm business enterprise belonging or run by this household?

○ Yes [1]
○ No [0]

[ls_work_help_farm] select_one

4.5 In the last 7 days, has ${em_name} helped (for at least one hour) in an agricultural activity or cared for livestock belonging or run by this household?

○ Yes [1]
○ No [0]

[ls_work_intern] select_one

4.6 In the last 7 days, has ${em_name} worked (at least one hour) as an intern or an apprentice?

○ Yes [1]
○ No [0]

[ls_work_vol] select_one

4.7 In the last 7 days, has ${em_name} worked (at least one hour) as a volunteer?

○ Yes [1]
○ No [0]

[ls_npaid] select_one

4.8 Even though ${em_name} did not do any of these activities in the last 7 days: does he/she have a paid job that he/she would definitely return to?

○ Yes [1]
○ No [0]

[ls_nbiz] select_one

4.9 Even though ${em_name} did not do any of these activities in the last 7 days: does he/she have a non-farm business that he/she would definitely return to?

○ Yes [1]
○ No [0]

[ls_nfarm] select_one

4.10 Even though ${em_name} did not do any of these activities in the last 7 days:does he/she have an own or family farming activity that he/she would definitely return to?

○ Yes [1]
○ No [0]

[ls_nunpaid] select_one

4.11 Even though ${em_name} did not do any of these activities in the last 7 days: does he/she have an unpaid job that he/she would definitely return to?

○ Yes [1]
○ No [0]

[ls_napp] select_one

4.12 Even though ${em_name} did not do any of these activities in the last 7 days:does he/she have an unpaid apprenticeship or internship that he/she would definitely return to?

○ Yes [1]
○ No [0]

[ls_nvol] select_one

4.13 Even though ${em_name} did not do any of these activities in the last 7 days: does he/she have a volunteer activity that he/she would definitely return to?

○ Yes [1]
○ No [0]

[ls_abs_reason] select_multiple

4.14 Why was ${em_name} absent from work during the last 7 days?

□ Vacation, holidays [1]
□ Annual leave [2]
□ Illness, injury or temporary disability [3]
□ Maternity or paternity leave [4]
□ Temporary slack work for technical or economic reasons [5]
□ Strike or labor dispute [6]
□ Off season [7]
□ Lack of transport [8]
□ Movement restrictions due to COVID-19 [9]
□ Education or training [10]
□ Family or community responsibilities [11]
□ Closure due to COVID-19 [12]
□ Closure (not related to COVID-19) [13]
□ Temporary closure due to COVID-19 [14]
□ Temporary closure (not related to COVID-19) [15]
□ Waiting to attend interview [16]
□ Other (specify) [-96]
□ Don't know [-98]

[ls_abs_reason_sp] skip logic=[selected(${ls_abs_reason}, '-96')] text

4.15 Please specify the other reasons ${em_name} was absent from work during the last 7 days.

[ls_abs_contract] select_one

4.16 Does ${em_name} have an agreement or contract to return to the same job after their absence? or if it is their own/family business, is the business still operating?

○ Yes [1]
○ No [0]
○ Don't Know [-98]

[ls_abs_dur_return] select_one

4.17 After how long will ${em_name} return to work?

- ○ Less than 1 month [1]
- ○ 1 month to less than 3 months [2]
- ○ 3 months and above [3]
- ○ Not sure when returning [-98]
- ○ Not returning [5]

[ls_abs_income] select_one

4.18 Does ${em_name} (continue to) receive an income from his/her job during this absence?

- ○ Yes [1]
- ○ No [0]
- ○ Don't Know [-98]

[ls_primary_activities_status] select_one

4.19 What is the status of ${em_name}'s primary activty in terms of time?

- ○ Paid employee (outside HH) [1]
- ○ Paid employee (within HH) [2]
- ○ Working employer [3]
- ○ Own-account worker [4]
- ○ Members of producers’ cooperatives [5]
- ○ Contributing family worker [6]
- ○ Apprentice/intern [7]
- ○ Volunteer [8]
- ○ Other (Please specify) [-96]
- ○ Don't know [-98]

[ls_primary_activities_status_duty] text

4.19a What are your main tasks or duties?

[ls_primary_occupation] select_one

4.20 What kind of work does ${em_name} usually do in this job or business?

- ○ Managers [1]
- ○ Professional [2]
- ○ Technicians and associate professionals [3]
- ○ Clerical support workers [4]
- ○ Service and sales workers [5]
- ○ Skilled agricultural, forestry and fishery workers [6]
- ○ Craft and related trades workers [7]
- ○ Plant and machine operators, and assemblers [8]
- ○ Elementary Occupations [9]
- ○ Other (Please specify) [-96]
- ○ Don't know [-98]

[ls_primary_occupation_sp] skip logic=[${ls_primary_occupation}='-96'] text

4.21 Please specify other type of occupation.

[ls_primary_industry] select_one

4.22 What kind of economic activity is ${em_name}'s primary activity connected with?

○ Agriculture/Livestock/Forestry/Fishing [1]
○ Mining [2]
○ Manufacturing/Handicraft [3]
○ Construction [4]
○ Wholesale or Retail Trade [5]
○ Services [6]
○ Other (specify) [-96]
○ Don't know [-98]

[ls_primary_industry_sp] skip logic=[${ls_primary_industry}='-96'] text

4.23 Please specify other type of industry.

[ls_primary_employer] select_one

4.24 Who was ${em_name}'s main employer for the primary activity?

○ Private Sector Enterprise (Registered/Formal Sector) [1]
○ County Governments [2]
○ Central Government [3]
○ Teachers Service Commission (TSC) [4]
○ State Owned Enterprise [5]
○ United Nations Agency [6]
○ International NGOs [7]
○ Local NGO / CBO [8]
○ Faith based Organization [9]
○ Self employed - Formal [10]
○ Informal Sector (Employed) [11]
○ Informal Sector (Self-Employed) [12]
○ Small Scale Agriculture (Employed) [13]
○ Small Scale Agriculture (Self-Employed) [14]
○ Pastoralist Activities (Employed) [15]
○ Pastoralist Activities (Self-Employed) [16]
○ Cooperative / Community Group [17]
○ Other [-96]
○ Don’t Know [-98]

[ls_primary_employer_sp] skip logic=[${ls_primary_employer}='-96'] text

4.25 Please specify other type of employer.

[wrkseek_1] select_multiple

4.26 In the past 4 weeks what actions has ${em_name} taken to look for a job or start any kind of business/income generating activity?

□ None [0]
□ Registered or waited at employment agency [1]
□ Registered a dispute [2]
□ Placed or answered job advertisements [3]
□ Enquired at workplaces, farms, factories or [4]
□ Called on other possible employers [5]
□ Sought assistance from relatives or friends [6]
□ Waited at the street side or other place where casual workers are found [7]
□ Applied for permit to start business [8]
□ Applied for a loan from Microfinance [9]
□ Applied for a loan from a Savings Group [10]
□ Applied for a loan from a bank [11]
□ Sought financial assistance from friends or family members [12]
□ Sought a loan from a church or mosque [13]
□ Purchased land, a building, or equipment [14]
□ Looked at job advertisements [15]
□ Looked for land, a building, or equipment [16]
□ Other (specify) [98]

[wrkseek_1_sp] skip logic=[selected(${wrkseek_1},'98')] text

4.27 Please specify if other

[ls_no_seek] skip logic=[selected(${wrkseek_1},'0')] select_one

4.28 What is the main reason ${em_name} was not working or not looking for work in the past 4 weeks?

○ COVID-19 restrictions (specify) [-95]
○ No jobs available in the area [1]
○ Unable to work (incapacitated) [2]
○ Unable to find work requiring his/her skills [3]
○ Home maker (housewife/family responsibilities) [4]
○ Discouraged worker [5]
○ Child care problems [6]
○ Employers think too old / too young to work [7]
○ Full time student / pupil [8]
○ Awaiting the season for work [9]
○ Waiting to be recalled to former job [10]
○ Have already found a job which will start later [11]
○ Transportation problems [12]
○ Pregnancy [13]
○ Sickness / injury [14]
○ Don’t need work [15]
○ Business closed [16]
○ Retired [17]
○ Waiting to attend interview [18]
○ Other (specify) [-96]
○ Don’t know [-98]

[ls_no_seek_sp] skip logic=[${ls_no_seek}='-96' or ${ls_no_seek}='-95'] text

4.29 Please specify the reason that ${em_name} was not working or looking for work in the past 4 weeks?

[ls_search_employment] select_one

4.30 In order to earn income, are you looking for employment or self-employment opportunities ?

○ Employment [1]
○ Self-employment [2]

[ls_search_obstacles] skip logic=[${ls_search_employment}=1] select_one

4.31 What do you think is the main obstacle that ${em_name} faces in securing a job (i.e. employment)?

○ Lack of or inadequate skills [1]
○ Lack of information about the local labor market [2]
○ Lack of family/clan or political connections [3]
○ Language barrier [4]
○ Disability / chronic illness [5]
○ Lack of work permit [6]
○ Lack of legal documentation requirements (KRA PIN, Business License, Company Certificate) [7]
○ Lack of refugee documentation requirements (Refugee ID) [8]
○ Lack of other documentation [9]
○ Limited or irregular work opportunities [10]
○ Lack of work opportunities [11]
○ Conflict and insecurity in area of residence [12]
○ Other (Specify) [-96]
○ Don't know [-98]

[ls_search_obstacles_sp] skip logic=[${ls_search_obstacles}='-96'] text

4.32 Please specify the main obstacle that ${em_name} faces in securing a job:

[ls_search_support] skip logic=[${ls_search_employment}=1] select_one

4.33 What is the main support ${em_name} needs to secure a job (i.e. employment)?

○ Technical / Vocational skills training [1]
○ Continue / complete education [2]
○ Support with access to markets [3]
○ Securing contacts with other employers [6]
○ Securing work permit [7]
○ Securing other documentation [8]
○ Other (Specify) [-96]
○ Don't know [-98]

[ls_search_support_sp] skip logic=[${ls_search_support}='-96'] text

4.34 Please specify the main support that ${em_name} needs to secure a job (i.e. employment):

[ls_search_obstacles2] skip logic=[${ls_search_employment}=2] select_one

4.35 What do you think is the main obstacle that ${em_name} faces in securing self-employment/starting a business?

○ Lack of or inadequate skills [1]
○ Lack of information about the local labor market [2]
○ Lack of family/clan or political connections [3]
○ Language barrier [4]
○ Disability / chronic illness [5]
○ Lack of work permit [6]
○ Lack of legal documentation requirements (KRA PIN, Business License, Company Certificate) [7]
○ Lack of refugee documentation requirements (Refugee ID) [8]
○ Lack of other documentation [9]
○ Conflict and insecurity in area of residence [10]
○ Lack of start-up capital (seed capital) [11]
○ Lack of expansion capital [12]
○ Lack of access to loans or micro-credit [13]
○ Other (specify) [-96]
○ Don't know [-99]

[ls_search_obstacles_sp2] skip logic=[${ls_search_obstacles}='-96'] text

4.36 Please specify the main obstacle that ${em_name} face in securing self-employment/ starting a business:

[ls_search_support2] skip logic=[${ls_search_employment}=2] select_one

4.37 What is the main support ${em_name} needs to secure self-employment/starting a business?

○ Technical or vocational skills training [1]
○ Continue or complete education [2]
○ Business training [3]
○ Loan or credit [4]
○ Support with access to markets [5]
○ Securing work permit [6]
○ Securing other documentation [7]
○ Securing areas for agriculture [8]
○ Securing livestock / tools for agriculture [9]
○ Other (specify) [-96]
○ Don't know [-99]

[ls_search_support_sp2] skip logic=[${ls_search_support}='-96' or ${ls_search_support}='7'] text

4.38 Please specify the main support that ${em_name} needs to secure self-employment/starting a business:

[ls_displacement_activity] select_one

4.39 What kind of work did ${em_name} do before displacement?

○ None [0]
○ Paid employee [1]
○ Own-account worker in a non-agricultural business [2]
○ Own-account worker in an agricultural business [3]
○ Not working (seeking employment) [4]
○ Full time student / pupil [5]
○ Volunteer [8]
○ Unpaid housework [6]
○ Retired / income recipient [7]
○ Other (specify) [-96]
○ Don't know [-98]

[ls_displacement_activity_sp] skip logic=[${ls_displacement_activity}='-96'] text

4.40 Please specify other work:

[ls_skills_work_internet] select_one

4.41 How is ${em_name}'s knowledge and proficiency in using the internet? (search information, use email, use online maps)

○ Excellent [1]
○ Good [2]
○ Little [3]
○ Poor [4]
○ None [5]

[le_skills_computers] select_one

4.42 How is ${em_name}'s knowledge and proficiency in using computers (especially Microsoft Word and Excel)?

○ Excellent [1]
○ Good [2]
○ Little [3]
○ Poor [4]
○ None [5]

[le_skills_math] select_one

4.43 How is ${em_name}'s knowledge and proficiency in BASIC math (addition, subtraction, multiplication and division)?

○ Excellent [1]
○ Good [2]
○ Little [3]
○ Poor [4]
○ None [5]

[le_skills_math_advanced] select_one

4.44 How is ${em_name}'s knowledge and proficiency in advanced math (fractions, percentages, decimals)?

○ Excellent [1]
○ Good [2]
○ Little [3]
○ Poor [4]
○ None [5]

[le_skills_desired] select_one

4.45 What is the main skill you want to develop in order to earn decent living?

○ Small business management [1]
○ IT training [2]
○ Automobile mechanics [3]
○ Driving [4]
○ Skills in the food and catering sector [5]
○ Parlor workshop/Hair cutting [6]
○ Training for electrical work [7]
○ Skills in the construction industry (Masonry, Carpenter, Foreman) [8]
○ Professional skills [9]
○ Fashion designing skills (e.g. dressmaking) [10]
○ language skills (e.g. english, swahili) [11]
○ Other (specify) [-96]
○ Don't know [-98]

[le_skills_desired_sp] skip logic=[${le_skills_desired}='-96'] text

4.46 Please specify if other

REPEAT ENDS: MEMBER

REPEAT ENDS: FAMILY

SECTION 5: HOUSING CHARACTERISTICS

SECTION 5: HOUSING CHARACTERISTICS

THIS SECTION INCLUDES QUESTIONS RELATED TO THE HOUSEHOLD'S CHARACTERISTICS AND AMENITIES.

[hca_du] integer

5.1 How many dwelling units or shelters does this household occupy?

[hca_hr] integer

5.2 In total, how many habitable rooms does this household occupy across all dwelling units?

[hca_type] select_one

5.3 What kind of housing do you live in ?

○ Apartment of my own/with family [1]
○ Shared/Rented Apartment [2]
○ Run---down or unfinished shelter [3]
○ No home, staying temporarily with friends [4]
○ NGO/State-run shelter [5]
○ Homeless [6]

[hca_floor] select_one

5.4 What is the main material of the floor of this dwelling unit?

○ Earth / Sand [1]
○ Dung [2]
○ Wood planks [3]
○ Palm / Bamboo [4]
○ Finished floor parquet or polished wood [5]
○ Vinyl or asphalt strips [6]
○ Ceramic tiles [7]
○ Cement [8]
○ Carpet [9]
○ Other (Specify) [-96]
○ Don’t Know [-98]

[hca_floor_spec] skip logic=[${hca_floor}=-96] text

5.5 Please specify the floor material of this dwelling unit.

[hca_roof] select_one

5.6 What is the main roof material of the main dwelling unit?

○ Grass / Thatch / Makuti [1]
○ Dung / Mud [2]
○ Plastic or tent material [3]
○ Corrugated iron sheets [4]
○ Tin Cans [5]
○ Asbestos sheet [6]
○ Concrete [7]
○ Tiles [8]
○ Other (Specify) [-96]
○ Don’t Know [-98]

[hca_roof_spec] skip logic=[${hca_roof}=-96] text

5.7 Please specify the roof material of this dwelling unit.

[hca_wall] select_one

5.8 What is the main wall material of the main dwelling unit?

○ No Walls [1]
○ Cane / Palm / Trunks [2]
○ Plastic or tent material [3]
○ Grass / Reeds [4]
○ Mud [5]
○ Bamboo with mud [7]
○ Stone with mud [8]
○ Covered adode [9]
○ Plywood [10]
○ Cardboard [11]
○ Reused wood [12]
○ Corrugated iron sheets [13]
○ Cement [14]
○ Stone with lime / cement [15]
○ Bricks [16]
○ Cement blocks [17]
○ Wood planks / Shingles [18]
○ Other (Specify) [-96]
○ Don’t Know [-98]

[hca_wall_spec] skip logic=[${hca_wall}=-96] text

5.9 Please specify the wall material of this dwelling unit.

[hca_water] select_one

5.10 What is the main source of drinking water for your household?

○ Piped into dwelling [1]
○ Piped into plot / yard [2]
○ Public tap / Stand pipe [3]
○ Tubewell / Borehole with pump [4]
○ Protected well [5]
○ Unprotected well [6]
○ Protected spring [7]
○ Unprotected spring [8]
○ Rain water collection [9]
○ Tankers truck [10]
○ Cart with small tank / drum / buckets [11]
○ Bicycles with buckets [12]
○ River / Stream / Pond / Dam / Lake / Canal [13]
○ Bottled Water [14]
○ Other (Specify) [-96]
○ Don’t Know [-98]

[hca_water_spec] skip logic=[${hca_water}=-96] text

5.11 Please specify the main source of drinking water for your household.

[hca_water_suf] select_one

5.12 In the last month, has there been any time when your household did not have sufficient quantities of drinking water when needed?

○ No, always sufficient [0]
○ Yes, at least once [1]
○ Don’t Know [-98]

[hca_toilet] select_one

5.13 What kind of toilet facility does your household usually use?

○ Flush to piped sewer system [11]
○ Flush to septic tank [12]
○ Flush to pit latrine [13]
○ Flush to somewhere else [14]
○ Flush to unknown place / not sure [15]
○ Ventilated Improved Pit latrine (VIP) [21]
○ Pit latrine with slab [22]
○ Pit latrine without slab / Open pit [23]
○ Composting toilet [31]
○ Bucket toilet [41]
○ Hanging toilet / Hanging latrine [51]
○ No facility / Bush / Field [61]
○ Other (Specify) [-96]
○ Don’t Know [-98]

[hca_toilet_spec] skip logic=[${hca_toilet}=-96] text

5.14 Please specify what kind of toilet facility your household usually uses.

[hca_toilet_share] skip logic=[${hca_toilet}!=61 and ${hca_toilet}!=-98 and ${hca_toilet}!=-99] select_one

5.15 Do you SHARE this toilet with others who are not members of your household?

○ Yes [1]
○ No [0]

[hca_lighting] select_one

5.16 What is the main source of energy for lighting?

○ Electricity Mains [1]
○ Generator [2]
○ Fire [3]
○ Solar Energy [4]
○ Paraffin / pressure lamp [5]
○ Gas lamp [6]
○ Battery lamp [7]
○ Telephone light [8]
○ Torch (not from the phone) [9]
○ Candles [10]
○ Biogas lantern [11]
○ Other (Specify) [-96]
○ None [-97]
○ Don’t Know [-98]

[hca_lighting_spec] skip logic=[${hca_lighting}=-96] text

5.17 Please specify the main source of energy for lighting?

[hca_cooking] select_one

5.18 What is the main source of energy for cooking?

- ○ Collected firewood [1]
- ○ Purchased firewood [2]
- ○ Electricity [3]
- ○ LPG [4]
- ○ Natural gas [5]
- ○ Biogas [6]
- ○ Kerosene [7]
- ○ Coal / Lignite [8]
- ○ Charcoal [9]
- ○ Straw / Shrubs / Grass [10]
- ○ Animal / Dung [11]
- ○ Crop Residue [12]
- ○ Other (Specify) [-96]
- ○ Don’t Know [-98]

[hca_cooking_spec] skip logic=[${hca_cooking}=-96] text

5.19 Please specify the main source of energy for cooking?

SECTION 6: ASSETS

SECTION 6: ASSETS

THIS SECTION INCLUDES QUESTIONS RELATED TO THE HOUSEHOLD'S ASSETS.

Do you or anyone in your household own:

[hca_radio] select_one

6.1 A radio?

- ○ Yes [1]
- ○ No [0]

[hca_tv] select_one

6.2 A television?

- ○ Yes [1]
- ○ No [0]

[hca_satelite] select_one

6.3 A satellite dish?

- ○ Yes [1]
- ○ No [0]

[hca_cell] select_one

6.4 A smartphone?

- ○ Yes [1]
- ○ No [0]

[hca_fridge] select_one

6.5 A refrigerator?

○ Yes [1]
○ No [0]

[hca_table] select_one

6.6 A table?

○ Yes [1]
○ No [0]

[hca_bed] select_one

6.7 A bed? (wood or metal)

○ Yes [1]
○ No [0]

[hca_mattress] select_one

6.8 A mattress?

○ Yes [1]
○ No [0]

[hca_mosquitonet] select_one

6.9 A mosquito net?

○ Yes [1]
○ No [0]

[hca_fan] select_one

6.10 A fan?

○ Yes [1]
○ No [0]

[hca_bike] select_one

6.11 A bicycle? (owned, not borrowed)

○ Yes [1]
○ No [0]

[hca_motorbike] select_one

6.12 A motorcycle or tuk tuk? (owned, not borrowed)

○ Yes [1]
○ No [0]

[hca_car] select_one

6.13 A car?

○ Yes [1]
○ No [0]

[hca_generator] select_one

6.14 A generator?

○ Yes [1]
○ No [0]

[hca_solar] select_one

6.15 A solar panels? (separate from lighting source)

○ Yes [1]
○ No [0]

[hca_kerosenestove] select_one

6.16 A kerosene stove?

○ Yes [1]
○ No [0]

[hca_charcoaljiko] select_one

6.17 A charcoal jiko?

○ Yes [1]
○ No [0]

[hca_wheelbarrow] select_one

6.18 A wheelbarrow?

○ Yes [1]
○ No [0]

[hca_fencing] select_one

6.19 Corregated iron fencing?

○ Yes [1]
○ No [0]

[hca_animals] select_one

6.20 Chickens, ducks or other animals?

○ Yes [1]
○ No [0]

SECTION 7: ACCESS

L

SECTION 7: ACCESS

THIS SECTION INCLUDES QUESTIONS RELATED TO THE HOUSEHOLD'S ACCESS TO VARIOUS LIVELIHOODS ASSETS.

[resettled] select_one

7.1 Do you have nuclear family members that have been resettled to the United States, Europe or another high income country?

○ Yes [1]
○ No [0]

[resettled] select_one

7.2 Do you have friends or other relatives that have been resettled to the United States, Europe or another high income country?

○ Yes [1]
○ No [0]

[outsidecamp] select_one

7.3 Do you have friends or relatives in Kenya, outside your residence area?

○ Yes [1]
○ No [0]

[outsidecamp_where] skip logic=[${outsidecamp}=1] select_multiple

7.4 Where?

□ Kakuma camp [1]
□ Kalobeyei settlement [2]
□ Dadaab camp [3]
□ Other (specify): [99]

[outsidecamp_where_sp] skip logic=[selected(${outsidecamp_where},"99")] select_one

7.5 Please specify where:

○ Mombasa [1]
○ Kwale [2]
○ Kilifi [3]
○ TaRiver [4]
○ Lamu [5]
○ Taita/Taveta [6]
○ Garissa [7]
○ Wajir [8]
○ Mandera [9]
○ Marsabit [10]
○ Isiolo [11]
○ Meru [12]
○ Tharaka-Nithi [13]
○ Embu [14]
○ Kitui [15]
○ Machakos [16]
○ Makueni [17]
○ Nyandarua [18]
○ Nyeri [19]
○ Kirinyaga [20]
○ Murang'a [21]
○ Kiambu [22]
○ Turkana [23]
○ WePokot [24]
○ Samburu [25]
○ TraNzoia [26]
○ UasGishu [27]
○ Elgeyo/Marakwet [28]
○ Nandi [29]
○ Baringo [30]
○ Laikipia [31]
○ Nakuru [32]
○ Narok [33]
○ Kajiado [34]
○ Kericho [35]
○ Bomet [36]
○ Kakamega [37]
○ Vihiga [38]
○ Bungoma [39]
○ Busia [40]
○ Siaya [41]
○ Kisumu [42]
○ HoBay [43]
○ Migori [44]
○ Kisii [45]
○ Nyamira [46]
○ Nairobi City [47]

[remitance] select_one

7.6 In the past 12 months, have you ever received remittances from abroad?

○ Yes [1]
○ No [0]

[account] select_one

7.7 Do you, either by yourself or together with someone else, currently have an account at a bank or SACCO?

○ Yes [1]
○ No [0]

[accont_type] skip logic=[${account}=1] select_one

7.8 If you have an account with someone else, is the other person a refugee or a Kenyan?

○ It's my own account [1]
○ With a refugee [2]
○ With a Kenyan [3]

[service_type] skip logic=[${account}=1] select_multiple

7.9 What bank or service do you use?

□ SACCO [1]
□ Equity Bank Kenya [2]
□ Standard Chartered Bank Kenya [3]
□ Barclays Bank Kenya [4]
□ Kenya Commercial Bank [5]
□ Cooperative Bank of Kenya [6]
□ Diamond Trust Bank of Kenya [7]
□ National Bank of Kenya [8]
□ Stanbic Bank [9]
□ Commercial Bank of Africa [10]
□ NIK Bank [11]
□ Other (specify) [99]

[service_type_sp] skip logic=[selected(${service_type},'99')] text

7.10 Please specify :

[account_mobile] select_one

7.11 Do you, either by yourself or together with someone else, currently have an account for mobile banking?

○ Yes [1]
○ No [0]

[accont_mobile_type] skip logic=[${account_mobile}=1] select_one

7.12 If you have an account with someone else, is the other person a refugee or a Kenyan?

○ It's my own account [1]
○ With a refugee [2]
○ With a Kenyan [3]

[loan] select_multiple

7.13 In the past 12 months, have you, by yourself or together with someone else, borrowed any money from any of the following sources?

□ From a bank or another type of FORMAL financial institution [1]
□ From community savings group or other INFORMAL financial institution [2]
□ From family, relatives, or friends [3]
□ No [0]

[saving_type] select_multiple

7.14 What kind of saving practices do you engage in?

- □ Village/Community Savings and Loans associations [1]
- □ Bank Savings [2]
- □ Mpesa/Mshwari Saving/Digital banking [3]
- □ Pillow banking [99]
- □ None [0]

[saving_type_sp] skip logic=[selected(${service_type},"99")] text

7.15 Please specify :

[saving_support] select_multiple

7.16 What kind of support do you require to formalize your saving practices?

- □ Refugee documentation to open a bank account [1]
- □ Assistance to join a VSLA/ CSLA [2]
- □ Access to Sim Card and Mobile Money [3]
- □ Other (specify) [99]

[saving_support_sp] skip logic=[selected(${saving_support},"99")] text

7.17 Please specify :

[insurance] select_one

7.18 Have you ever received or purchased insurance of any kind?

- ○ Yes [1]
- ○ No [0]

[insurance_current] skip logic=[${insurance}=1] select_one

7.19 Do you currently hold an insurance?

- ○ Yes [1]
- ○ No [0]

[insurance_national] select_one

7.20 Are you enrolled in the National Hospital Insurance Fund (NHIF)?

- ○ Yes [1]
- ○ No [0]

[insurance_national_card] skip logic=[${insurance_national}=1] select_one

7.21 Do you have an NHIF card?

- ○ Yes [1]
- ○ No [0]

[aware_nhif] skip logic=[${insurance_national}=0] select_one

7.22 If no, are you aware of the NHIF?

- ○ Yes [1]
- ○ No [0]

[reason_not_enrolled_nhif] skip logic=[${aware_nhif}=1] select_one

7.23 Why you have not enrolled?

○ Insufficient funds [1]
○ Need money to meet basic needs [2]
○ Other, please specify [99]

[reason_not_enrolled_nhif_sp] skip logic=[${reason_not_enrolled_nhif}=99] text

7.24 Please specify :

[self_help_group] select_one

7.25 Are you a member of a CBO/Self Help Group (Chamas)?

○ Yes [1]
○ No [0]

SECTION 8: VULNERABILITIES

SECTION 8: VULNERABILITIES

THIS SECTION INCLUDES QUESTIONS RELATED TO THE VULNERABILITY OF THE FAMILY MEMBERS.

REPEAT STARTS: FAMILY

REPEAT STARTS: MEMBER

START THE QUESTIONS ABOUT:

${vul_name}

THE FOLLOWING QUESTIONS ASK ABOUT DIFFICULTIES ${vul_name} MAY HAVE DOING CERTAIN ACTIVITIES BECAUSE OF A HEALTH PROBLEM.

[vul_documentation] select_multiple

8.1 Please indicate the documentation available to ${vul_name}:

□ Birth certificate [1]
□ Passport from country of origin [2]
□ Refugee identity card [3]
□ Convention travel document [4]
□ Movement pass (to travel outside the camp) [5]
□ Kenyan work permit [6]
□ School diploma [7]
□ No documents [8]
□ Other (Please specify) [-96]
□ Don't know [-98]

[vul_documentation_sp] skip logic=[selected(${vul_documentation}, '-96')] text

8.2 Please specify the other type of documentation available to ${vul_name}:

[ds_bd] select_one

8.3 Does ${vul_name} have difficulty seeing,?

○ No – no difficulty [1]
○ Yes – some difficulty [2]
○ Yes – a lot of difficulty [3]
○ Cannot do at all [4]
○ Don't know [-98]
○ Refused to respond [-99]

[ds_df] select_one

8.4 Does ${vul_name} have difficulty hearing?

○ No – no difficulty [1]
○ Yes – some difficulty [2]
○ Yes – a lot of difficulty [3]
○ Cannot do at all [4]
○ Don't know [-98]
○ Refused to respond [-99]

[ds_pms_1] select_one

8.5 Does ${vul_name} have difficulty walking or climbing steps?

○ No – no difficulty [1]
○ Yes – some difficulty [2]
○ Yes – a lot of difficulty [3]
○ Cannot do at all [4]
○ Don't know [-98]
○ Refused to respond [-99]

[ds_mm] select_one

8.6 Does ${vul_name} have difficulty remembering or concentrating?

○ No – no difficulty [1]
○ Yes – some difficulty [2]
○ Yes – a lot of difficulty [3]
○ Cannot do at all [4]
○ Don't know [-98]
○ Refused to respond [-99]

[ds_pms_2] select_one

8.7 Does ${vul_name} have difficulty (with self-care such as) washing all over or dressing?

○ No – no difficulty [1]
○ Yes – some difficulty [2]
○ Yes – a lot of difficulty [3]
○ Cannot do at all [4]
○ Don't know [-98]
○ Refused to respond [-99]

[ds_sd] select_one

8.8 Using his/her usual (customary) language, does ${vul_name} have difficulty communicating, for example understanding or being understood?

○ No – no difficulty [1]
○ Yes – some difficulty [2]
○ Yes – a lot of difficulty [3]
○ Cannot do at all [4]
○ Don't know [-98]
○ Refused to respond [-99]

[ds_bd_aid] skip logic=[${ds_bd}>1] select_one

8.9 Does ${vul_name} wear glasses?

○ Yes [1]
○ No [0]

[ds_df_aid] skip logic=[${ds_df}>1] select_one

8.10 Does ${vul_name} use a hearing aid?

○ Yes [1]
○ No [0]

REPEAT ENDS:

REPEAT ENDS:

[ds_special_needs] select_multiple

8.11 Do you have any of the following as members of your household who live with you?

□ Separated child (meaning, child is not part of nuclear family) [1]
□ Disabled persons (Physical) [2]
□ Disabled persons (Cognitive) [3]
□ Disabled persons (sensory disability) [4]
□ Older persons (above 60 years old) [5]
□ Pregnant woman or nursing mother [6]
□ Persons with serious medical condition(s) [7]
□ None of these [99]

SECTION 9: SOCIAL COHESION

L

SECTION9: SOCIAL COHESION

THIS SECTION INCLUDES QUESTIONS RELATED TO SOCIAL COHESION.

READ THE FOLLOWING TO THE RESPONDENT.

I am going to read you a series of statements regarding your social life and how safe you feel in general. For each statement, please tell me if you (i) strongly agree, (ii) agree, (iii) neither agree nor disagree, (iv) disagree, or (v) strongly disagree.

[trust_community] select_one

9.1 Generally speaking, do you feel that people in this neighborhood are trustworthy?

○ Strongly agree [1]
○ Agree [2]
○ Neither agree nor disagree [3]
○ Disagree [4]
○ Strongly disagree [5]
○ Don't Know [-98]
○ Refused to respond [-99]

[trust_hcommunity] select_one

9.2 Generally speaking, do you feel that most people in the host community are trustworthy?

○ Strongly agree [1]
○ Agree [2]
○ Neither agree nor disagree [3]
○ Disagree [4]
○ Strongly disagree [5]
○ Don't Know [-98]
○ Refused to respond [-99]

[safe_hcommunity] select_one

9.3 Would you feel safe if you went to City Centre by yourself?

○ Strongly agree [1]
○ Agree [2]
○ Neither agree nor disagree [3]
○ Disagree [4]
○ Strongly disagree [5]
○ Don't Know [-98]
○ Refused to respond [-99]

[more_intraction] select_one

9.4 Would you feel comfortable if your child or grandchild were to socialize or be friends with children of host community people?

○ Strongly agree [1]
○ Agree [2]
○ Neither agree nor disagree [3]
○ Disagree [4]
○ Strongly disagree [5]
○ Don't Know [-98]
○ Refused to respond [-99]

[safe_day] select_one

9.5 Do you feel safe walking alone in your area/neighbourhood during the day?

○ Strongly agree [1]
○ Agree [2]
○ Neither agree nor disagree [3]
○ Disagree [4]
○ Strongly disagree [5]
○ Don't Know [-98]
○ Refused to respond [-99]

[safe_night] select_one

9.6 Do you feel safe walking alone in your area/neighbourhood at night?

- ○ Strongly agree [1]
- ○ Agree [2]
- ○ Neither agree nor disagree [3]
- ○ Disagree [4]
- ○ Strongly disagree [5]
- ○ Don't Know [-98]
- ○ Refused to respond [-99]

[crime_common] select_one

9.7 Are crimes common in your neighborhood/areas?

- ○ Strongly agree [1]
- ○ Agree [2]
- ○ Neither agree nor disagree [3]
- ○ Disagree [4]
- ○ Strongly disagree [5]
- ○ Don't Know [-98]
- ○ Refused to respond [-99]

[participation_stucture] select_one

9.8 Do you feel that you are able to express your opinion through the existing community leadership structure?

- ○ Strongly agree [1]
- ○ Agree [2]
- ○ Neither agree nor disagree [3]
- ○ Disagree [4]
- ○ Strongly disagree [5]
- ○ Don't Know [-98]
- ○ Refused to respond [-99]

[participation_consideration] select_one

9.9 Do you feel like your opinion is being considered for decisions which affect your well-being?

- ○ Strongly agree [1]
- ○ Agree [2]
- ○ Neither agree nor disagree [3]
- ○ Disagree [4]
- ○ Strongly disagree [5]
- ○ Don't Know [-98]
- ○ Refused to respond [-99]

[participation_national] select_one

9.10 How much would you say the political system in Kenya allows people like you to have a say in what the government does?

○ Strongly agree [1]
○ Agree [2]
○ Neither agree nor disagree [3]
○ Disagree [4]
○ Strongly disagree [5]
○ Don't Know [-98]
○ Refused to respond [-99]

[interact_social] select_one

9.11 in the last 7 days, did any member of this household interact with an individual from a neighboring community in any way?

○ Yes [1]
○ No [0]

SECTION 10: COPING MECHANISM

L

SECTION 10: COPING MECHANISM

THIS SECTION INCLUDES QUESTIONS ABOUT HOW THE HOUSEHOLD COPES WITH DIFFICULTIES.

[no_food] select_one

10.1 During the PAST 30 DAYS, how often was there no food to eat of any kind in your house because of lack of resources to buy food?

○ Never [1]
○ Rarely (1-2 times) [2]
○ Sometimes (3-5 times) [3]
○ Often (more than 5 times) [4]
○ Don't know [-98]
○ Refused to respond [-99]

[coping_multiple] select_multiple

10.2 During the PAST 30 DAYS, did you or anyone in your household use any of these strategies to cope with the lack of food or lack of money to buy food?

□ Sold households assets/goods e.g., radio, furniture, refrigerator, television, jewelry etc. [1]
□ Reduced spending on health or education [2]
□ Sold productive assets or means of transport e.g., sewing machines, wheel barrows, etc. [3]
□ Spent savings [4]
□ Borrowed money / food from a formal lender or bank [5]
□ Sold a house or land [7]
□ Withdrew children from school [8]
□ Sold last female animal [9]
□ Begged [10]
□ Sold more animals than usual [11]
□ Other (please specify) [99]
□ None [0]
□ Don't know [-98]
□ Refused to respond [-99]

[coping_multiple_sp] skip logic=[selected(${coping_multiple},'99')] text

10.3 Please specify if other

[coping_lackfood] select_one

10.4 In the past 7 days, were there times when your household did not have enough food or enough money to buy sufficient food?

- ○ Yes [1]
- ○ No [0]

[coping_lackfood_frq] skip logic=[${coping_lackfood}=1] select_one

10.5 How many days?

- ○ 1 day [1]
- ○ 2 days [2]
- ○ 3 days [3]
- ○ 4 days [4]
- ○ 5 days [5]
- ○ 6 days [6]
- ○ 7 days [7]
- ○ Don't know [-98]
- ○ Refugsed to respond [-99]

[coping_less_food] select_one

10.6 Over the last 7 days, were there times when your household rely on less preferred and less expensive food?

- ○ Yes [1]
- ○ No [0]

[coping_less_food_frq] skip logic=[${coping_less_food}=1] select_one

10.7 How many days?

- ○ 1 day [1]
- ○ 2 days [2]
- ○ 3 days [3]
- ○ 4 days [4]
- ○ 5 days [5]
- ○ 6 days [6]
- ○ 7 days [7]
- ○ Don't know [-98]
- ○ Refugsed to respond [-99]

[coping_borrowfood] select_one

10.8 Over the last 7 days, were there days when your household borrow food or money for food from friends or relatives?

- ○ Yes [1]
- ○ No [0]

[coping_borrowfood_frq] skip logic=[${coping_borrowfood}=1] select_one

10.9 How many days?

○ 1 day [1]
○ 2 days [2]
○ 3 days [3]
○ 4 days [4]
○ 5 days [5]
○ 6 days [6]
○ 7 days [7]
○ Don't know [-98]
○ Refugsed to respond [-99]

[coping_limitportion] select_one

10.10 Over the last 7 days, were there days when your household limit portion size at mealtimes for all household members?

○ Yes [1]
○ No [0]

[coping_limitportion_frq] skip logic=[${coping_limitportion}=1] select_one

10.11 How many days?

○ 1 day [1]
○ 2 days [2]
○ 3 days [3]
○ 4 days [4]
○ 5 days [5]
○ 6 days [6]
○ 7 days [7]
○ Don't know [-98]
○ Refugsed to respond [-99]

[coping_restrictcom] select_one

10.12 Over the last 7 days, were there days when your household restrict consumption by adults in order for small children to eat?

○ Yes [1]
○ No [0]

[coping_restrictcom_frq] skip logic=[${coping_restrictcom}=1] select_one

10.13 How many days?

○ 1 day [1]
○ 2 days [2]
○ 3 days [3]
○ 4 days [4]
○ 5 days [5]
○ 6 days [6]
○ 7 days [7]
○ Don't know [-98]
○ Refugsed to respond [-99]

[coping_reducemeal] select_one

10.14 Over the last 7 days, were there days when your household reduce the number of meals eaten in a day for all household members?

○ Yes [1]
○ No [0]

[coping_reducemeal_frq] skip logic=[${coping_reducemeal}=1] select_one

10.15 How many days?

○ 1 day [1]
○ 2 days [2]
○ 3 days [3]
○ 4 days [4]
○ 5 days [5]
○ 6 days [6]
○ 7 days [7]
○ Don't know [-98]
○ Refugsed to respond [-99]

SECTION 11: TRAJECTORIES AND INTENTIONS TO MOVE

L

SECTION 11: TRAJECTORIES AND INTENTIONS TO MOVE

THIS SECTION INCLUDES QUESTIONS ABOUT HOW THE HOUSEHOLD WAS DISPLACED AND FORESEES THEIR POTENTIAL RETURN / RELOCATION WITHIN KENYA/THIRD COUNTRY SOLUTIONS.

[displacement_reasion] select_multiple

11.1 What are the main reasons why your household left your country of origin?

□ COVID-19 [0]
□ Lack of safety in my village [1]
□ Lack of safety in neighboring villages [2]
□ Increased crime, Risks to safety and insecurity but not lack of safety [3]
□ Drought/famine/flood [4]
□ Lack of access to home/area of housing / area of livelihood/livestock [5]
□ Lack of access to education services and health services [6]
□ Lack of employment opportunities [7]
□ Death of husband/family reasons [8]
□ Other reasons (specify) [99]
□ Don't know [-98]
□ Refused to respond [-99]

[displacement_reasion_other] skip logic=[selected(${displacement_reasion},"99")] text

11.2 Please specify other reason:

[return] select_one

11.3 Do you want to leave this location at some point in time?

○ Yes [1]
○ No [0]

[return_where] skip logic=[${return}=1] select_one

11.4 Do you plan to return to your original place of residence or go to a new location?

○ I plan to return to my original place of residence / country of origin [1]
○ I plan to go to a new area [2]
○ Don't know [99]
○ Refused to respond [-99]

[return_when] skip logic=[${return_where}=1 or ${return_where}=2] select_one

11.5 When do you plan to leave?

○ In the next 3 months [1]
○ 3 months to 1 year [2]
○ More than one year [3]
○ Don’t know yet [-98]

[leave_future_plan] skip logic=[${return_where}=1 or ${return_where}=2] select_one

11.6 To which place would you like to move?

○ I plan to move to Kakuma refugee camp [1]
○ I plan to move to Dadaab refugee camp [2]
○ I plan to move to Kalobeyei Settlement [3]
○ I plan to return to my country of origin but to a new location there [4]
○ I plan to return to my country of origin in the same location I lived before [5]
○ I plan to go to a new country [6]
○ Don’t know / not applicable [-98]
○ Refused to respond [-99]

[leave_future_region] skip logic=[${leave_future_plan}=6] select_one

11.7 To what region would you like to move?

○ Africa [1]
○ Asia (inlcuding Australia) [2]
○ Europe [3]
○ North America [4]
○ Central America [5]
○ South America [6]
○ Other (specify) [96]

[leave_future_region_sp] skip logic=[${leave_future_region}=96] text

11.7a Please specify.

[leave_future_work_leave] skip logic=[${leave_future_plan}=6] select_one

11.8 If you and the household members get the formal right to settle freely and live and work in Kenya, would you and the household members still want to move to a new country?

○ Yes [1]
○ No [0]

[leave_reasion] skip logic=[${return}=1] select_multiple

11.9 Thinking about the place where you live now, what are the main reasons why you or members of your household want to move?

□ Increased crime, violence and insecurity but not armed conflict in the area [3]
□ Fear of ethnic/political/religious discrimination and persecutions [4]
□ Drought/famine/flood [5]
□ Lack of access to home/land/livestock [6]
□ Lack of access to education services and health services [7]
□ Lack of employment opportunities [8]
□ No family here anymore / family reasons [9]
□ Lack of humanitarian assistance (particularly food) [10]
□ Lack of proper management of the site/ site is crowded [11]
□ Tensions with the host community [12]
□ Uncertain legal situation in the country [13]
□ Other reasons (specify) [99]
□ Don't know / not applicable [-98]

[leave_reasion_other] skip logic=[selected(${leave_reasion},"99")] text

11.10 Please specify other reason:

[not_movebackreasion] skip logic=[${leave_future_plan}=1 or ${leave_future_plan}=2 or ${leave_future_ select_multiple

11.11 Thinking about your place of origin, what are the main reasons why you or members of your household don’t want to move back?

□ Armed conflict in the area of origin [1]
□ Armed conflict in the surrounding areas [2]
□ Increased crime, violence and insecurity but not armed conflict in the area [3]
□ Fear of ethnic/political/religious discrimination and persecutions [4]
□ Drought/famine/flood [5]
□ Lack of access to home/land/livestock [6]
□ Lack of access to education services and health services [7]
□ Lack of employment opportunities [8]
□ No family there anymore / family reasons [9]
□ Will lose access to humanitarian aid [10]
□ Other reasons (specify) [99]
□ Don't know / not applicable [-98]
□ Refused to respond [-99]

[not_movebackreasion_other] skip logic=[selected(${not_movebackreasion},"99")] text

11.12 Please specify other reason:

[foreseeable_plan] skip logic=[${leave_future_plan}=1 or ${leave_future_plan}=2 or ${leave_future_ select_one

11.13 Since you do not want to return to the home country, what do you plan in the foreseeable future?

○ Stay in urban area [1]
○ Move from urban area to refugee camp and stay there [4]
○ Seek solution in third country [5]
○ Don't know [-98]
○ Refused to respond [-99]

[not_stay_reasion] skip logic=[${leave_future_plan}=1 or ${leave_future_plan}=2 or ${leave_future_ select_multiple

11.14 Thinking about the place where you or the household members would go, what are the main reasons why you and members of your household want to move?

□ Better security there [1]
□ Better access to home/land/livestock [2]
□ Better access to education and health services [3]
□ Better access to livelihood/employment opportunities [4]
□ To live with family or community members / family reasons [5]
□ Access to humanitarian aid (particularly food and water) [6]
□ Other reasons (specify) [99]
□ Don't know [-98]
□ Refused to respond [-99]

[not_stay_reasion_other] skip logic=[selected(${not_stay_reasion},"99")] text

11.15 Please specify other reason:

[stay_reasion] skip logic=[${return}=0] select_multiple

11.16 Thinking about the place where you live now, what are the main reasons why you or members of your household want to stay?

□ Better security here [1]
□ Better access to home/area of housing and area of livelihood/livestock [2]
□ Better access to education and health services [3]
□ Better access to livelihood/employment opportunities [4]
□ To continue living with family or community members /family reasons [5]
□ Access to humanitarian aid (particularly food and water) [6]
□ Other reasons (specify) [99]
□ Don't know / not applicable [-98]

[stay_reasion_other] skip logic=[selected(${stay_reasion},"99")] text

11.17 Please specify other reason:

[not_move_reasion] skip logic=[${return}=0] select_multiple

11.18 Thinking about the place where you would go if you needed to leave Kenya, what are the main reasons why you don't want to move?

□ Armed conflict in the area [1]
□ Armed conflict in surrounding areas [2]
□ Increased crime, violence and insecurity but not armed conflict in the area [3]
□ Fear of ethnic/political/religious discrimination and persecutions [4]
□ Drought/famine/flood [5]
□ Lack of access to [6]
□ home/land/livestock [7]
□ Lack of access to education services and health services [8]
□ Lack of employment opportunities [9]
□ No family there anymore / family reasons [10]
□ Will lose access to humanitarian aid [11]
□ Other reasons (specify) [99]
□ Don't know / not applicable [-98]

[not_move_reasion_other] skip logic=[selected(${not_move_reasion},"99")] text

11.19 Please specify other reason:

[info_source] select_one

11.20 What is your main source of obtaining information about the situation in your country of origin?

○ Radio [1]
○ TV [2]
○ Internet and social media [3]
○ Written for example [4]
○ Newspapers/magazines/ bulletins (pamphlets, brochure) [5]
○ Community leaders/elders [6]
○ Family and friends [7]
○ Religious leaders [8]
○ Politicians [9]
○ Charities, NGO, UN or other public organizations [10]
○ Site Management Committees [11]
○ Other (specify) [99]
○ Don't know [-98]
○ Refused to respond [-99]

[info_source_other] skip logic=[${info_source}=99] text

11.21 Specify other source of information:

[all_information_YN] select_one

11.22 Does your household have all the information they need in order to make a decision whether to return to your place of origin, move to some new place, or remain here?

○ Yes [1]
○ No [0]

[wanted_info] skip logic=[${all_information_YN}=0] select_multiple

11.23 What are the main types of information your household wants that you are not currently receiving in order to make an informed decision whether to return to your place of origin, move to some new place, or remain here?

□ Information about political situation in the country of origin [1]
□ Information about political situation in Kenya regarding refugees [2]
□ Information about security [3]
□ situation in the country of origin [4]
□ Information about security situation on transit routes [5]
□ Information about availability of basic services (food, water, shelter, education, health, etc.) [6]
□ Information about quality of basic services [7]
□ Information about availability of work and livelihood opportunities [8]
□ Information about access to land/ property/ housing [9]
□ Information about transport options [10]
□ Information about whether the household will we be able to return if we leave the camp [11]
□ Information about whether the household will have access to humanitarian aid on return [12]
□ Information about how to obtain or renew documents (identity card, birth certificate etc.) [13]
□ Other (specify) [99]
□ Don't know [-98]
□ Refused to respond [-99]

[wanted_info_other] skip logic=[selected(${wanted_info},"99")] text

11.24 Specify other source of information:

[action_alternative] select_one

11.25 What will you do if you face economic difficulties in the urban area and UNHCR and its partner are not able to provide you with assistance?

○ Relocation to refugee camp [1]
○ Return to home country [2]
○ Will try to find work in the urban [3]
○ Will make arrangements with my communities [4]
○ Other (specify) [99]

[action_alternative_other] skip logic=[${action_alternative}=99] text

11.26 Specify other:

[interview_s] skip logic=[${phone}!=1 and ${phone}!=2] select_one

11.27 WHAT IS THE STATUS OF THIS INTERVIEW?

○ Completed [1]
○ Incomplete - Scheduled an appointment [2]
○ Incomplete, no adult present [-95]
○ Incomplete, absent [-96]
○ Incomplete - Respondent Refuses [-97]
○ Incomplete - Moved to another place [-98]

[Feedbacks] skip logic=[${phone}!=1 and ${phone}!=2] text

11.28 READ THE FOLLOWING TO THE RESPONDENT:Is there any other information you would like to share with me?

[Enumerator_Notes] text

11.29 ENUMERATOR'S NOTE (IF ANY)

THIS IS THE END OF THE ASSESSMENT.

PLEASE MARK THE FORM AS FINALISED, AND CLICK ON "SAVE FORM AND EXIT" IN THE NEXT SCREEN.
